# Comparative effectiveness and cost-effectiveness of cardioprotective glucose-lowering therapies for type 2 diabetes in Brazil: a Bayesian network model

**DOI:** 10.1186/s13561-023-00466-3

**Published:** 2023-10-25

**Authors:** Ana Claudia Cavalcante Nogueira, Joaquim Barreto, Filipe A. Moura, Beatriz Luchiari, Abrão Abuhab, Isabella Bonilha, Wilson Nadruz, J. Michael Gaziano, Thomas Gaziano, Luiz Sergio F. de Carvalho, Andrei C. Sposito, on behalf of the Brazilian Heart Study Group

**Affiliations:** 1 Cardiology Division, Unicamp Medical School, São Paulo, SP Brazil; 2 Escola Superior de Ciências da Saúde (ESCS), Brasília, Distrito Federal Brazil; 3 TIMI Study Group, Division of Cardiovascular Medicine, Brigham and Women’s Hospital, Harvard Medical School, Boston, MA USA; 4 Heart Institute (InCor), Do Hospital das Clínicas - FMUSP, Sao Paulo, Brazil; 5 Department of Medicine, Harvard Medical School, Boston, MA USA; 6 Department of Cardiovascular Medicine, Brigham & Women’s Hospital, Boston, MA USA; 7 Clarity Healthcare Intelligence, Jundiaí, SP Brasil; 8 Atherosclerosis and Vascular Biology Laboratory (Atherolab), State University of Campinas (Unicamp), Sao Paulo, Campinas 13084-971 Brazil

**Keywords:** Pioglitazone, SGLT2i, GLP1-RA, Cost-effectiveness, Glucose-lowering therapy, Cardiovascular disease

## Abstract

**Background:**

The escalating prevalence of type 2 diabetes (T2DM) poses an unparalleled economic catastrophe to developing countries. Cardiovascular diseases remain the primary source of costs among individuals with T2DM, incurring expenses for medications, hospitalizations, and surgical interventions. Compelling evidence suggests that the risk of cardiovascular outcomes can be reduced by three classes of glucose-lowering therapies (GLT), including SGLT2i, GLP-1A, and pioglitazone. However, an evidence-based and cost-effective protocol is still unavailable for many countries. The objective of the current study is to compare the effectiveness and cost-effectiveness of GLT in individuals with T2DM in Brazil.

**Methods:**

We employed Bayesian Networks to calculate the incremental cost-effectiveness ratios (ICER), expressed in international dollars (Int$) per disease-adjusted life years [DALYs] averted. To determine the effectiveness of GLT, we conducted a systematic review with network meta-analysis (NMA) to provide insights for our model. Additionally, we obtained cardiovascular outcome incidence data from two real-world cohorts comprising 851 and 1337 patients in primary and secondary prevention, respectively. Our cost analysis took into account the perspective of the Brazilian public health system, and all values were converted to Int$.

**Results:**

In the NMA, SGLT2i [HR: 0.81 (95% CI 0.69–0.96)], GLP-1A [HR: 0.79 (95% CI 0.67–0.94)], and pioglitazone [HR: 0.73 (95% CI 0.59–0.91)] demonstrated reduced relative risks of non-fatal cardiovascular events. In the context of primary prevention, pioglitazone yielded 0.2339 DALYs averted, with an ICER of Int$7,082 (95% CI 4,521–10,770) per DALY averted when compared to standard care. SGLT2i and GLP-1A also increased effectiveness, resulting in 0.261 and 0.259 DALYs averted, respectively, but with higher ICERs of Int$12,061 (95% CI: 7,227–18,121) and Int$29,119 (95% CI: 23,811–35,367) per DALY averted. In the secondary prevention scenario, all three classes of treatments were deemed cost-effective at a maximum willingness-to-pay threshold of Int$26,700. Notably, pioglitazone consistently exhibited the highest probability of being cost-effective in both scenarios.

**Conclusions:**

In Brazil, pioglitazone presented a higher probability of being cost-effective both in primary and secondary prevention, followed by SGLT2i and GLP-1A. Our findings support the use of cost-effectiveness models to build optimized and hierarchical therapeutic strategy in the management of T2DM.

**Trial registration:**

CRD42020194415.

**Supplementary Information:**

The online version contains supplementary material available at 10.1186/s13561-023-00466-3.

## Background

The global prevalence of Type 2 Diabetes Mellitus (T2DM) is on a relentless rise, foreshadowing an unprecedented economic and social burden on a global scale. It is anticipated that international economic spending on T2DM will surge to around US$2.1 trillion by 2030 [1]. Currently, the majority of this financial burden is shouldered by low- and middle-income countries, where 80% of adults with T2DM reside, and where access to therapeutic resources remains limited [2].

Cardiovascular diseases (CVD) continue to be the primary source of expenses for individuals with T2DM, incurring costs related to medications, hospitalizations, and surgical interventions [3, 4]. There is compelling evidence indicating that three classes of glucose-lowering therapies can reduce the risk of cardiovascular and/or cardiorenal outcomes. For instance, sodium-glucose transporter-2 inhibitors (SGLT2i) lower the risk of cardiovascular death, heart failure, and kidney events. Similarly, glucagon-like peptide 1 receptor agonists (GLP-1A) primarily reduce the incidence of major cardiovascular events (MACE). Furthermore, there is a substantial body of data and literature supporting the effectiveness of pioglitazone, an older and more cost-effective drug class, in reducing MACE [5].

These therapies, however, differ not only in their mechanisms of action but also in various aspects, including their impact on different components of cardiovascular events, types of adverse events, and cost. This divergence underscores the need for a cost-effectiveness assessment to facilitate a better understanding and utilization of these drugs within a hierarchical therapeutic approach. Such an analysis is particularly vital in the context of low- and middle-income countries, where it can provide valuable guidance for future therapeutic strategies that are both feasible and comprehensive, considering the limitations of their healthcare resources.

To address this pressing issue, our study introduces a Bayesian network (BN) combined with a Markov influence diagram (MID). This innovative approach is designed to compare the cost-effectiveness of pioglitazone, SGLT2i, and GLP-1A against sulfonylureas in individuals with T2DM in Brazil. Both BN and MIDs are powerful probabilistic graphical models that enable transparent economic analyses, utilizing transition probabilities and disease prevalence data extracted from intricate healthcare scenarios [6].

To address this unmet need, in this study we created a Bayesian network (BN) with a Markov influence diagram (MID) aimed at comparing the cost-effectiveness of pioglitazone, SGLT2i, and GLP-1A against sulfonylureas in individuals with T2DM from Brazil. BN and MIDs are probabilistic graphical models that permit transparent economic analyses using transition probabilities and disease prevalence from complex healthcare scenarios [6]. To feed these models with essential data, we conducted a comprehensive systematic review and network meta-analysis to evaluate the effectiveness of these drugs in reducing cardiovascular events. Additionally, we examined the incidence of critical clinical endpoints in individuals with T2DM by drawing insights from two extensive real-world cohorts situated in Brazil.

## Methods

### Systematic review and meta-analysis to estimate the effectiveness of glucose-lowering therapies

#### Selection strategies

We conducted a systematic review following the established procedures for literature review and meta-analysis, adhering to the guidelines outlined in the Preferred Reporting Items for Systematic Reviews and Meta-Analyses (PRISMA) statement. Our meta-analysis is registered with PROSPERO under the registration number CRD42020194415. For the literature review, we meticulously searched various electronic databases, including Medline (PubMed), ClinicalTrials.gov, Cochrane Central Register of Controlled Trials, Embase, European Union Clinical Trials Register, and the World Health Organization (WHO).

**Fig. 1:**
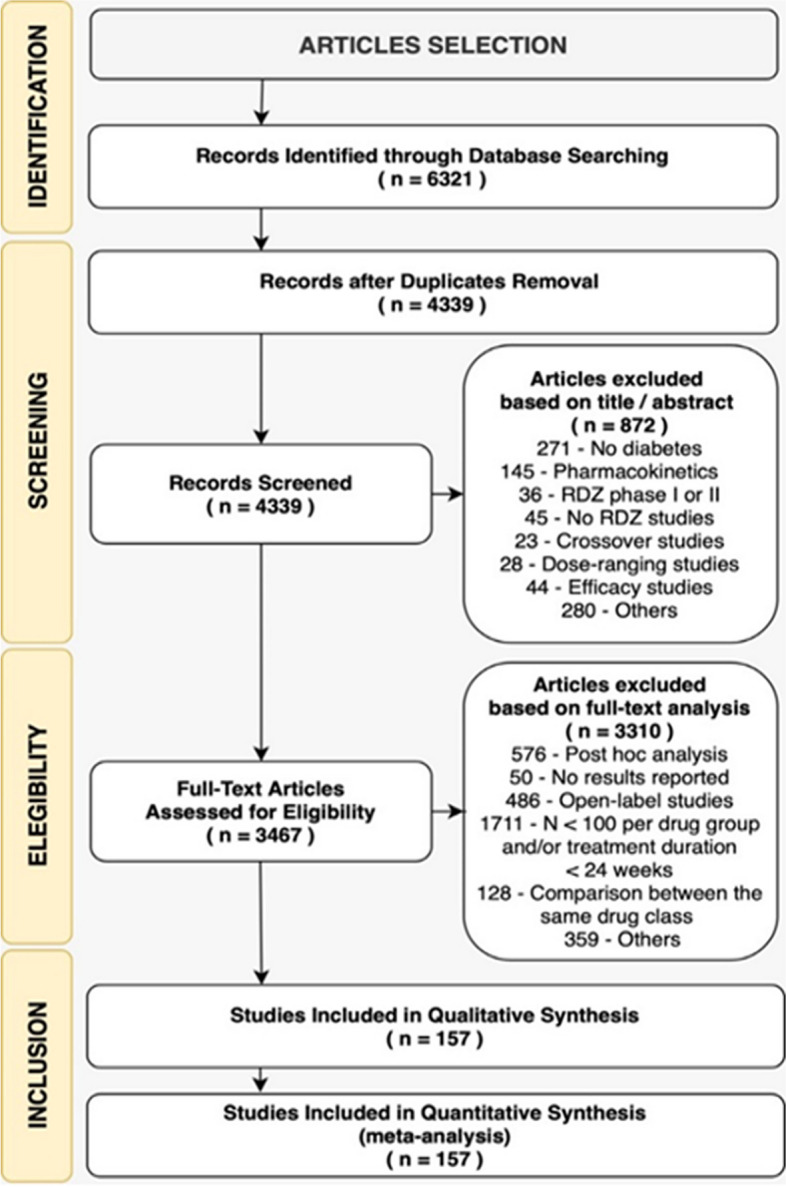
Flow diagram for RCT selection process in a systematic review

We conducted our search on the International Clinical Trials Registry Platform between April and August 2021. Our search terms included "anti-hyperglycemic drugs," "type 2 diabetes," "mortality," and "cardiovascular events". To ensure the quality and relevance of the studies included in our analysis, we established specific inclusion criteria (detailed in Supplementary material, Table S1). These criteria encompassed the following: (i) Randomized clinical trials (RCTs) involving subjects with T2DM; (ii) Inclusion of only articles published in English; (iii) Double-blind RCTs, preferably in phase 3 or 4; (iv) Studies with 100 or more patients per arm, a follow-up duration exceeding 24 weeks, and the reporting of pre-specified endpoints, including death from any cause, MACE, and hospitalization for heart failure (HHF) (refer to Fig. 1). As we anticipated that major cardiovascular outcome trials would possess sufficient statistical power to discern the cardiovascular benefits or risks associated with different glucose-lowering therapies, we predefined in the PROSPERO protocol that we would exclusively consider RCTs with more than 100 patients per arm.

#### Data extraction and quality assessment

Initially, the data extracted from the studies were input into a Microsoft Excel® spreadsheet by five authors (A.C.C.N., I.B., J.B., and B.L.), with any discrepancies addressed through discussion with the senior researchers (A.C.S. and L.S.F.C.). The extracted data encompassed a range of key information, including: (i) first author's name; (ii) year of publication; (iii) sample size; (iv) duration of follow-up; (v) patient characteristics (such as sex, age, race); (vi) duration of diabetes; (vii) active (or experimental) and comparative drugs; (viii) history of cardiovascular events; (ix) history of heart failure; (x) average systolic and diastolic blood pressure; (xi) weight; (xii) body mass index (BMI); (xiii) hemoglobin A1c (HbA1c); (xiv) estimated glomerular filtration rate (eGFR); (xv) clinical outcomes. This comprehensive dataset served as the foundation for our analysis.

#### Data synthesis and statistical analyses

The primary endpoint focused on non-fatal MACE, specifically defined as non-fatal myocardial infarction and non-fatal stroke. Secondary endpoints encompassed: (i) Hospitalizations related to heart failure (HHF); (ii) All-cause mortality. We present dichotomous variables as percentages and report continuous variables as either mean ± SD or median with the interquartile range. Baseline data were derived through weighted calculations. For our analysis, we employed a comprehensive network meta-analysis (CNMA) framework, facilitating an indirect comparison of the study drugs. To assess the potential impact of these therapies on clinical outcomes, we calculated hazard ratios (HRs) using a random-effects CNMA model based on the combined data from the trials.

To assess statistical heterogeneity among the trials, we utilized the I [2] statistic, along with its associated 95% confidence intervals. This statistic, derived from Cochran's Q as [100 × (Q–df ÷ Q)], offers insight into the extent of variation attributable to differences between the trials. For the evaluation of potential publication bias, we constructed funnel charts and conducted the Egger test. A two-tailed p-value below 0.05 was considered indicative of statistical significance in determining the treatment effect. Our data analysis was carried out using R v4.0.1 (2020, Auckland, New Zealand) in conjunction with the *discomb, metaviz,* and *metafor* packages.

### Clinical data for estimating transition probabilities and disease prevalence

We utilized two prospective longitudinal cohorts of patients with T2DM to provide the transition probabilities for the Markov Influence Diagram (MID). These cohorts are as follows:

1. The Brazilian Diabetes Study (BDS) (NCT04949152): This study comprised diabetic outpatient subjects admitted to the Clinical Research Center Outpatient Clinic at the State University of Campinas (Campinas, Brazil) from July 2016 to July 2019. The BDS included a total of 1,030 individuals, with 851 in the primary prevention category. The median follow-up period was 2.2 years (interquartile range [IQR] 0.9), as detailed in Supplementary Material, Table S5.
2. The Brasilia Cardiovascular Registry for Quality of Care and Outcomes (B-CaRe:QCO): This cohort consisted of diabetic individuals admitted due to acute coronary syndrome (ACS) across all public hospitals in Brasilia, Brazil, spanning from January 2013 to January 2019. The B-CaRe:QCO enrolled a total of 1,158 individuals with T2DM, who were followed for a median of 5.1 years (IQR 3.2), as outlined in Supplementary material, Table S6.

These cohorts provided valuable data for our analysis of transition probabilities within the MID.

#### Standard therapy

We defined standard glucose-lowering therapy as the median treatment regimen commonly prescribed to individuals with T2DM. This standard treatment encompasses all therapies typically utilized in Brazil, as detailed in Supplementary Table S5 and S6. This comprehensive approach takes into account the proportion of individuals using metformin, DPP4 inhibitors, sulfonylureas, and insulin as part of their treatment regimen.

#### Model description

To estimate treatment outcomes effectively, we designed a multi-state model incorporating Bayesian Networks (BN) and MID. This model takes into account the chronic and progressive nature of T2DM, recognizing that patients transition through various health states over the course of the disease. In a formal sense, a MID comprises a directed acyclic graph featuring three distinct sets of nodes: decision nodes, probability nodes, and utility nodes. Decision nodes represent actions within the control of decision-makers, while probability nodes signify uncertain events. In the context of medical applications, utility nodes reflect medical outcomes and costs, including factors like quality of life, morbidity, mortality, and economic costs [6]. Utilizing these MIDs, we can assign transition probabilities to the model and transform the structural relationships among variables into Markov cycles. This approach allows us to study the cost-effectiveness of interventions over a specific time horizon [6]. Both the BN and MID were constructed using OpenMarkov (CISIAD, Madrid, Spain) [6].

**Fig. 2:**
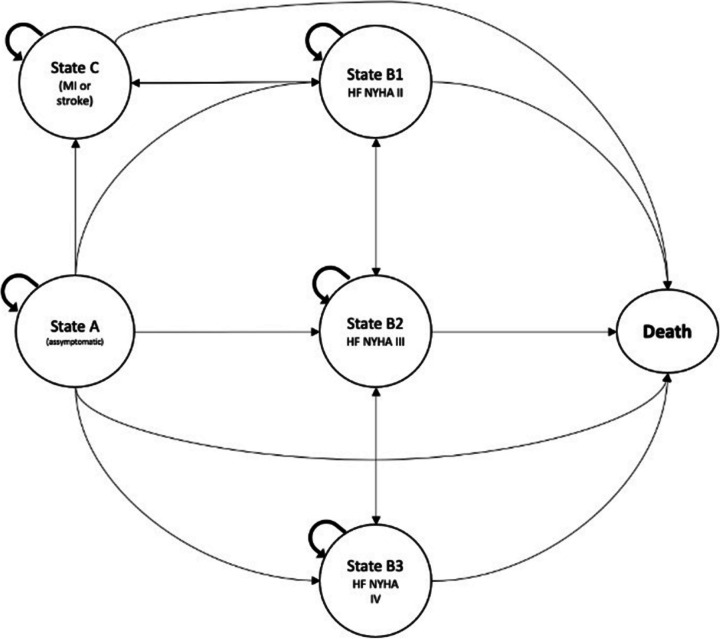
Multistage Markov Model built with Markov Influence Diagram and Bayesian Network

In this analysis, we defined four distinct patient states that capture potential effects on cardiovascular disease: (i) Stage A: Asymptomatic; (ii) Stages B1, B2, and B3: Heart failure functional classes II, III, and IV, respectively; (iii) Stage C: Non-fatal myocardial infarction and non-fatal stroke; (iv) Stage D: Death (Refer to Fig. 2).

We initiated our assessment with individuals diagnosed with T2DM either in the primary prevention phase (state A, with no individuals in states B or C at the outset) or in the secondary prevention phase of cardiovascular disease (state C, with no individuals in states A or B initially). This differentiation stems from the distinct benefits offered by therapies in primary versus secondary prevention scenarios.

It's important to note that we did not consider the possibility of individuals transitioning from a symptomatic heart failure state to an asymptomatic state. While it's conceivable for an individual with symptomatic heart failure (NYHA class II-IV) to become asymptomatic (NYHA class I), the therapies are typically not withdrawn as *per protocol*. Consequently, the general annual costs for individuals with heart failure who transition to NYHA class I remain similar to those in NYHA class II [7].

#### Effectiveness, transition probabilities, and mortality

In the primary prevention scenario, patients were assumed to initiate follow-up at the age of 47, which corresponds to the mean age at T2DM diagnosis in the BDS cohort (as indicated in Supplementary Table S5). The time horizon for this analysis extended to 25 years or 25 cycles. This cycle duration was selected based on the life expectancy of patients with T2DM in Brazil, which is approximately 72 years [8]. To address the challenge posed by limited data on the transition from states A to B in primary prevention, we divided the data from our meta-analysis into two subsets: (i) RCTs with follow-up durations of up to 8 years from T2DM diagnosis; (ii) RCTs with patients diagnosed with T2DM for more than 8 years. This division is grounded in the significant differences in heart failure incidence between individuals recently diagnosed with diabetes versus those with a longer history of the disease [9, 10].

In the secondary prevention scenario, individuals were assumed to commence follow-up at the age of 60, which corresponds to the mean age at the first cardiovascular or cerebrovascular event or hospitalization in the B-CaRe:QCOR cohort (as detailed in Supplementary Table S6). The time horizon for this analysis was set at 12 years or 12 cycles. Transition probabilities, as presented in Table 1, were derived from annual incidence rates of heart failure (HF), myocardial infarction/stroke (MI/stroke), and death (in patient-years). These rates were obtained from various sources, including our meta-analysis, the BDS cohort, the B-CaRe:QCOR cohort, diabetic individuals in the FIGHT trial [11], and diabetic individuals included in a meta-analysis by Vaduganathan et al [12]. The trials encompassed in Vaduganathan et al.'s meta-analysis included DELIVER [13], EMPEROR-Preserved [14], DAPA-HF [15], EMPEROR-Reduced [16], and SOLOIST-WHF [17].

**Table 1:** Transition probabilities of each treatment strategy

	Probability [< 8 years / ≥ 8 year from diagnosis of T2DM]
Stage A (asymptomatic) to Stage B (heart failure)	
Standard therapy^a^	0.0027 / 0.0161
Standard therapy + pioglitazone^a^	0.0039 / 0.0304
Standard therapy + SGLT2i^a^	0.0018 / 0.0109
Standard therapy + GLP-1A^a^	0.0024 / 0.0151
Stage A (asymptomatic) to Stage C (coronary artery disease or stroke)	
Standard therapy^b^	0.0078 / 0.0196
Standard therapy + pioglitazone^ba^	0.0050 / 0.0102
Standard therapy + SGLT2i^ba^	0.0059 / 0.0111
Standard therapy + GLP-1A^ba^	0.0057 / 0.0110
Stage A (asymptomatic) to Stage D (death)	
Standard therapy^b^	0.0029 / 0.0091
Standard therapy + pioglitazone^ba^	0.0027 / 0.0088
Standard therapy + SGLT2i^ba^	0.0022 / 0.0085
Standard therapy + GLP-1A^ba^	0.0021 / 0.0085
Stage B (heart failure) to Stage C (coronary artery disease or stroke)	
Standard therapy^de^	0.0811 / 0.1551
Standard therapy + pioglitazone	^(nru)^
Standard therapy + SGLT2i^d^	0.0800 / 0.1507
Standard therapy + GLP-1A^e^	0.0797 / 0.1510
Stage B (heart failure) to Stage D (death)	
Standard therapy^de^	0.1400 / 0.1502
Standard therapy + pioglitazone	^(nru)^
Standard therapy + SGLT2i^d^	0.1187 / 0.1328
Standard therapy + GLP-1A^e^	0.1319 / 0.1486
Stage C (coronary artery disease or stroke) to Stage B (heart failure)	
Standard therapy^c^	0.0466 / 0.0765
Standard therapy + pioglitazone^ca^	0.0707 / 0.0912
Standard therapy + SGLT2i^ca^	0.0331 / 0.0488
Standard therapy + GLP-1A^ca^	0.0490 / 0.0617
Stage C (coronary artery disease or stroke) to Stage D (death)	
Standard therapy^c^	0.0399 / 0.0618
Standard therapy + pioglitazone^ca^	0.0327 / 0.0498
Standard therapy + SGLT2i^ca^	0.0309 / 0.0480
Standard therapy + GLP-1A^ca^	0.0301 / 0.0478

^a^Present meta-analysis

^b^BDS cohort

^c^B-CaRe:QCOR cohort

^d^diabetic individuals in the meta-analysis by Vaduganathan et al [12], which included the trials: DELIVER [13], EMPEROR-Preserved [14], DAPA-HF [15], EMPEROR-Reduced [16] and SOLOIST-WHF [17]

^e^FIGHT trial [11]; nru: non-recommended use

We assumed that the probability of transition rates increases with age, with more pronounced differences observed between individuals with up to 8 years from the diagnosis of T2DM and those with over 8 years. To transform annual incidence rates of HF, MI/stroke, and death into probabilities and calculate the probability of transition over a specific time interval, we followed the formula recommended by Sonnenberg et al [18]. This formula incorporates the probability (p), the rate (r), and the time (t):

$$\mathrm p=1-e\;^{\text{-}rt}$$

$$\mathrm r=-\left[1\mathrm n\left(1-\mathrm p\right)\right]\;/\;\mathrm t$$

#### Costs

In our cost analyses, we adopted the perspective of the Brazilian Unified Health System (SUS) as the payer. The reimbursement amounts for cost items are uniform nationwide and are determined by the SUS price list (as outlined in Table 2).

**Table 2:** Mean cost for cardiovascular procedures and hospitalizations during the period from 2013 to 2019 in Brazilian SUS

	**Brazilian SUS (mean cost in Int$)**
*Outpatient care* (annual cost)	
Diabetes with no complications (Stage A)	2,018.00
Diabetes with heart failure (Stage B)	7,458.00
Diabetes with ACS or stroke (Stage C)	5,536.00
Haemodialysis (annual cost)	9,113.00
*Procedures* typically performed (per 1 procedure)	
PCI	6,328.00
CABG	12,655.00
*Intensive care unit* (ICU) *hospitalization* (per day)^a^	555.00
*In-patient care* by condition treated (per 1 hospitalization)	
Heart failure^a^	1,252.00
Diabetes^a^	497.00
Cardiac arrest^a^	2,000.00
Unstable angina^a^	1,112.00
Myocardial infarction^a^	1,778.00
Ischemic stroke^a^	3,321.00
Haemorrhagic stroke^b^	7,043.00

We used the reimbursement costs for *procedures*, *ICU hospitalizations* and *in-patient care* to estimate mean annual cost related to relevant clinical events (RECE) including hemodialysis and their relative incidences in patients-years observed in B-CaRe:QCO and BDS cohorts. RECE-related mean annual costs were as follows: Stage A (asymptomatic) = Int$ 1,083.00; Stage B (heart failure) = Int$ 1,278.00; Stage C (coronary artery disease or stroke) = Int$ 2,059.00; Stage D (dead) = Int$ 0.00

^a^Cost of hospitalization (includes lab and imaging exams and the cost for beds during average length of hospital stay), not including the costs for procedures

^b^Includes the costs for procedures and hospitalizations. Data for Brazil obtained from DATASUS (SIH/SUS and SIGTAP), the data processing system of the Brazilian Health Ministry

To provide costs in international terms, we initially obtained the monetary values from the SUS price list in Brazilian reais (R$). Subsequently, we converted these values into international dollars (Int$) using the purchasing power parity (PPP) factor for 2022, which was 2.53. This data extraction method from SUS databases has been previously detailed [19].

As the SUS does not encompass pioglitazone, SGLT2i, or GLP-1A within its array of anti-hyperglycemic medications, we calculated the combined cost by considering SUS expenditures along with out-of-pocket spending (OPS). OPS was approximated based on the average prices from 2 to 4 major pharmacy chains, adjusted for each drug's market share (as detailed in Supplemental Material, Table S5). These prices were updated as of September 6^th^, 2023. The total annual cost of anti-diabetic therapies, including both SUS and OPS, was estimated at R$ 1342.21 (Int$ 530.52). Subsequently, we determined the incremental cost of pioglitazone, SGLT2i, and GLP-1A based on the mean annual pharmacy prices (listed in Table 3). To ensure the accuracy of these values, we utilized the National Wide Consumer Price Index (IPCA), periodically reported by the Brazilian Institute of Geography and Statistics (IBGE), to adjust the reported prices to their current values. In the economic analysis, we considered direct medical costs, encompassing the resources directly utilized for a patient's treatment, such as medication expenses, diagnostic tests, hospitalizations, medical procedures, and follow-up. These direct health costs were estimated for each state and year throughout the study's duration, incorporating an annual discount rate of 5% for both costs and outcomes.

**Table 3:** Estimated compound SUS and out-of-pocket spending with anti-diabetic therapies (updated in September 6^th^ 2023)

	Mean monthly costs (Brazilian real – R$)								Mean annual costs (R$ or Int$)	
Drug class / Drug	Price 1	Price 2	Price 3	Price 4	Mean cost	sd	Market share-adjusted drug cost	Market share-adjusted drug class cost	Mean annual add-on drug class cost (R$)	Mean annual add-on drug class cost (Int$)
Biguanide								R$ 4,98		
Metformin 850 mg	R$ 2.10	R$ 2.01	R$ 3.49	R$ 2.67	R$ 2.57	R$ 0.68	R$ 4.98			
Sulfonylureas								R$ 7.66		
Glybenclamide 5 mg	R$ 0.99	R$ 1.05	R$ 0.63	R$ 0.63	R$ 0.82	R$ 0.23	R$ 0.36			
Glimepiride	R$ 78.39				R$ 78.39		R$ 3.14			
Gliclazide 30 mg	R$ 10.48	R$ 13.92	R$ 10.28		R$ 11.56	R$ 2.05	R$ 4.16			
Glitazone								R$ 2.68	511.92	202.34
Pioglitazone 30-45 mg	R$ 47.00	R$ 42.00	R$ 39.00		R$ 42.66	R$ 4.04	R$ 2.68			
GLP1 receptor agonists								R$ 28.50	8.505.54	3.361.87
Liraglutide	R$ 686.00	R$ 482.02	R$ 496.75		R$ 554.92	R$ 113.75	R$ 23.60			
Semaglutide	R$ 774.00	R$ 1017.00	R$ 797.00		R$ 862.67	R$ 134.15	R$ 4.90			
DPP4 inhibitors								R$ 21.13	2.304.84	911.00
Vildagliptin 50 mg	R$ 80.89	R$ 68.98	R$ 63.90		R$ 71.26	R$ 8.72	R$ 8.55			
Sitagliptin 50 mg	R$ 116.03	R$ 142.23			R$ 129.13	R$ 18.53	R$ 10.33			
Alogliptin 25	R$ 75.88	R$ 67.42	R$ 63.03		R$ 68.77	R$ 6.53				
Linagliptin 5 mg	R$ 195.25	R$ 254.06			R$ 224.66	R$ 41.59	R$ 2.25			
SGLT2 inhibitors								R$ 43.18		
Dapagliflozin 10 mg	R$ 99.80	R$ 95.60	R$ 147.99		R$ 114.46	R$ 29.11	R$ 17.86		1.373,52	542.89
Empagliflozin 25 mg	R$ 182.89	R$ 251.59	R$ 282.08	R$ 182.89	R$ 224.86	R$ 50.03	R$ 25.32			
Insulin										
Regular	R$ 21.00	R$ 23.12	R$ 23.76		R$ 22.63	R$ 1.45	R$ 1.13	R$ 1.13		
NPH	R$ 18.81	R$ 16.88	R$ 19.79		R$ 18.49	R$ 1.48	R$ 2.59	R$ 2.59		
Total monthly cost in reais (Brazilian real – R$)								111.85		
Total annual cost in reais (Brazilian real – R$)								1.342.21		
Total annual cost in purchase parity-adjusted Int$								530.52		

Purchase power parity-adjusted conversion: 1 Int$ = 2, 53 R$. Final model considered only the prices for Dapagliflozin as this iSGLT2 was included in a government-led access program that significantly dropped its prices

#### Cost-effectiveness ratio

The incremental cost-effectiveness ratio (ICER) was computed by taking the difference in total costs between the two treatment sequences and dividing it by the difference in their total effectiveness, quantified in disease-adjusted life years (DALYs) averted. In accordance with the World Health Organization's cost-effectiveness guidelines, ICERs are typically evaluated in relation to one to three times the per capita gross domestic product (GDP) of Brazil [20]. In our study, we established the maximum willingness to pay threshold (mWTPT) at Int$ 8,900 per DALY, which corresponds to the *per capita* GDP of Brazil. The results were presented in terms of the average duration of survival and the international dollars spent per DALY averted.

#### Sensitivity analysis

In our meta-analysis, we conducted sensitivity analyses aimed at assessing potential sources of bias. The first sensitivity analysis exclusively considered RCTs with a mean time since the diagnosis of T2DM of fewer than 8 years, followed by a second sensitivity analysis based on RCTs with a mean time of ≥ 8 years since diagnosis.

For the BN/MID model, we carried out a probabilistic sensitivity analysis to gauge the robustness of the model results concerning key parameters. This analysis encompassed variations in the effectiveness of each add-on therapy and changes in drug prices. To address cost uncertainties, we performed probabilistic sensitivity analysis using a 5% discount rate, aligning with the recommendation of the Brazilian Ministry of Health [21]. Additionally, we conducted one-way sensitivity analyses to assess the impact of each discount parameter.

## Results

### Systematic review and meta-analysis to estimate the effectiveness of glucose-lowering therapies

As of April 2021, our electronic search yielded 3,467 references after eliminating duplicates and those that did not meet our inclusion criteria based on title and abstract information. Following a meticulous evaluation against predetermined inclusion and exclusion criteria, we identified a total of 157 RCTs suitable for qualitative synthesis and meta-analysis. For a visual representation of the selection process and the study network, please refer to Fig. 1 in the Supplementary material, as well as Figure S1. These 157 RCTs collectively involved 267,508 patients distributed across 176 active arms, with an average follow-up duration of 1.46 years, resulting in a sample size of 684,389 patient-years. You can find baseline characteristics of the enrolled individuals within the trials in Supplementary material Table S2.

Importantly, all the studies demonstrated a low risk of bias as per the Cochrane Collaboration tool for assessing bias risk (Supplementary material, Table S3) and received a High-Quality rating from the GRADE system. Additionally, Supplementary material Table S4 and Figure S2 indicate the absence of significant publication bias in funnel plots, along with the absence of significant small study bias as determined by the Egger tests.

To calculate non-fatal MACE, we subtracted the number of cases with incident 3-p MACE (comprising cardiovascular death, non-fatal MI, and non-fatal stroke) from the total number of cardiovascular deaths. Consequently, we identified 105 RCTs with 118 active arms for this specific outcome, involving 52 exclusions. Among these exclusions, 34 RCTs did not align with our defined MACE criteria, and 18 RCTs failed to report the number of cardiovascular deaths.

In a network meta-analysis utilizing a random-effects model, we compared SGLT2i, GLP-1A, and pioglitazone with sulfonylureas. The results revealed a reduction in the relative risk of non-fatal MACE with hazard ratios (HR) of 0.81 (95% CI 0.69 to 0.96, *p* = 0.011), 0.79 (95% CI 0.67 to 0.94, *p* = 0.0039), and 0.73 (95% CI 0.59 to 0.91, *p* = 0.0057), respectively. Notably, no heterogeneity was observed (I2 = 0%, *p* = 0.984), as indicated in Supplementary material Figure S3. Furthermore, the meta-analysis produced consistent results with and without additive effects, with no significant difference noted (p for difference 0.58).

Data on all-cause mortality were reported in 140 RCTs, which encompassed 12,927 events. When compared to sulfonylureas, treatment with GLP-1A and SGLT2i yielded a reduction in the relative risk of death from all causes, with hazard ratios (HR) of 0.85 (95% CI 0.76 to 0.95, *p* = 0.0098) and 0.85 (95% CI 0.76 to 0.95, *p* = 0.0069), respectively. Importantly, there was no observed heterogeneity (I2 = 0%, *p* = 0.9987), as illustrated in Supplemental Material Figure S4. In the case of pioglitazone, it was associated with an HR of 0.91 (95% CI 0.74 to 1.13, *p* = 0.37) for all-cause mortality. Both the meta-analysis with and without additive effects yielded similar results, with no significant difference noted (p for difference 0.88).

The relative risk of HHF was solely reduced in patients treated with an SGLT2i (HR 0.67, 95% CI 0.54 to 0.82; *p* = 0.0003) compared to sulfonylurea. Conversely, pioglitazone treatment was associated with a higher risk of HHF (HR 1.44, 95% CI 1.16 to 1.79, *p* = 0.0004), and no heterogeneity was observed (I2 = 0%, *p* = 0.84), as depicted in Supplementary material Figure S5. Notably, the meta-analysis, whether conducted with or without additive effects, yielded consistent results (p for difference 0.84).

### Sensitivity analysis in the meta-analysis

In the first sensitivity analysis, we exclusively considered RCTs with a mean time since the diagnosis of T2DM of fewer than 8 years. Within this subset, the mean time since T2DM diagnosis averaged 4.67 ± 1.94 years. In this initial analysis, we observed non-fatal MACE at a rate of 8.18 ± 7.7 per 1,000 patients-years (across 39 treatment arms and 34 studies), all-cause deaths were documented at a rate of 6.94 ± 7.4 per 1,000 patients-years (across 53 treatment arms and 45 studies), and HHF occurred at a rate of 2.72 ± 4.2 per 1,000 patients-years (across 30 treatment arms and 22 studies). Within this subgroup of RCTs, we did not identify significant differences when comparing the study drugs to sulfonylureas in terms of non-fatal MACE, all-cause death, and HHF. This may be attributed to the low incidence of cardiovascular events, resulting in limited statistical power. However, it is noteworthy that the overall trends and magnitude toward a reduction in each outcome remained consistent, as observed in the main analyses. A second sensitivity analysis was conducted based on RCTs involving individuals with a mean duration of T2DM of ≥ 8 years, with the mean time since T2DM diagnosis measuring 10.61 ± 2.9 years within this subset.

In this sensitivity analysis, we observed non-fatal MACE at a rate of 10.73 ± 9.9 per 1,000 patients-years, involving 56 treatment arms and 48 studies. All-cause deaths were recorded at a rate of 10.22 ± 10.9 per 1,000 patients-years, across 62 treatment arms and 55 studies, while HHF occurred at a rate of 16.1 ± 18.5 per 1,000 patients-years, encompassing 27 treatment arms and 22 studies. Within the RCTs considered in this sensitivity analysis, no significant change in direction or magnitude was evident when evaluating the impact of anti-diabetic drugs compared to the findings from the general analyses. In comparison to sulfonylureas, the following trends were noted: (i) SGLT2i, GLP-1A, and pioglitazone reduced the incidence of non-fatal MACE, (ii) SGLT2i and GLP-1A reduced the incidence of all-cause death, and (iii) SGLT2i reduced the incidence of HHF, while pioglitazone increased the incidence of HHF compared to placebo. Notably, it's important to highlight that the magnitude of the effect for pioglitazone in this subgroup was consistently higher regarding the incidence of HHF, with an HR of 1.892 (95%CI 1.03–3.48, *p* = 0.0402, I2 = 0.221), as compared to the findings in the main analyses.

When comparing the incidences of all three outcomes among RCTs with a time length of < 8 years vs. ≥ 8 years, we observed significant differences only for HHF (2.72 ± 4.2 vs. 16.1 ± 18.5 per 1,000 patients-years, *p* = 0.002). We did not detect any significant interaction with the number of individuals in primary prevention. It's worth noting that the number of RCTs reporting the proportion of individuals in primary/secondary prevention was relatively low (*n* = 46 RCTs).

### Disease-adjusted life years (DALY) averted

**Fig. 3:**
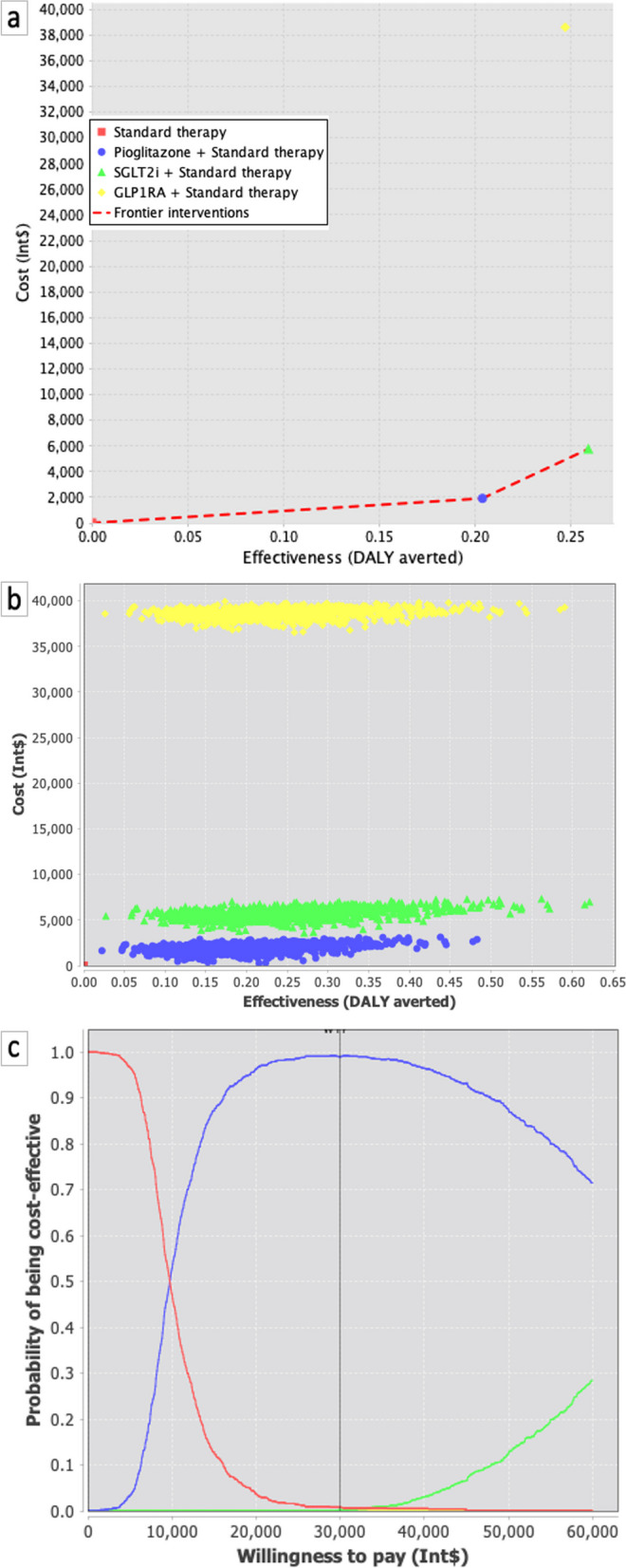
Cost-effectiveness planes for 25 cycles (25 years) departing from an asymptomatic, cardiovascular disease-free, and recently diagnosed T2DM at baseline with a mean age of 47 years-old (departing from state A). The model considered an age-dependent progressive increase in transition probabilities as described in Table 1. **a**. sensitivity analysis considering 5% discount rates for costs and effectiveness for each add-on therapy. **b**. cost-effectiveness chart comparing incremental cost and effectiveness of pioglitazone, SGLT2i, and GLP-1A on top of standard therapy. **c**. cost-effectiveness acceptability curve

Using the transition probabilities outlined in Table 1, we conducted MIDs considering transitions to states as described in Fig. 2. In the primary prevention scenario, the addition of pioglitazone was associated with a mean incremental effectiveness of 0.204 DALYs per patient compared to standard care (Fig. 3a). The mean incremental cost was estimated at Int$ 202.34 (95% CI: 187 to 234), primarily driven by the higher purchasing cost of the medication. The calculated ICER was Int$ 9,403 (95% CI: 6,272; 12,479) per DALY averted. In contrast, the addition of SGLT2i or GLP-1A resulted in more pronounced incremental effectiveness (0.259 and 0.247, respectively). However, the incremental costs associated with these therapies led to higher ICERs, specifically Int$ 22,242 (95% CI: 17,793; 27,020) and Int$ 156,231 (95% CI: 150,119; 162,761) per DALY averted, respectively.

**Fig. 4:**
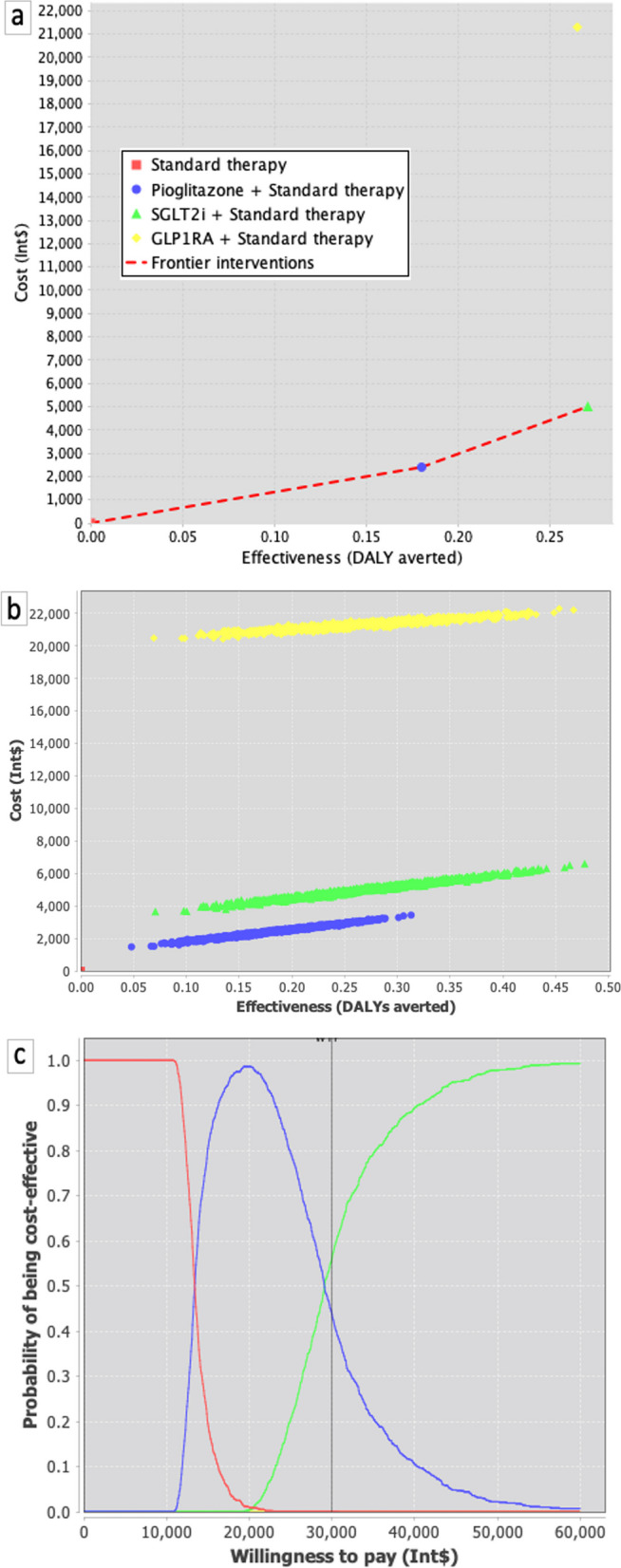
Cost-effectiveness planes for 12 cycles (12 years) departing from the age of 60 years-old, T2DM with mean 10 years since diagnosis and recent diagnosis of cardiovascular disease (departing from state C). The model considered an age-dependent progressive increase in transition probabilities as described in Table 1. **a**. sensitivity analysis considering 5% discount rates for costs and effectiveness for each add-on therapy. **b**. cost-effectiveness chart comparing incremental cost and effectiveness of pioglitazone, SGLT2i, and GLP-1A on top of standard therapy. **c**. cost-effectiveness acceptability curve

In the secondary prevention scenario (Fig. 4a), pioglitazone exhibited a mean incremental effectiveness of 0.180 DALYs per patient when compared to standard care. The ICER estimate was calculated at Int$ 13,319 (95% CI: 8,821; 18,293) per DALY averted. The addition of SGLT2i or GLP-1A resulted in more pronounced incremental effectiveness (0.271 and 0.265, respectively). However, the incremental costs associated with these therapies led to higher ICERs, specifically Int$ 18,471 (95% CI: 15,299; 21,340) and Int$ 80,355 (95% CI: 75,961; 84,966) per DALY averted, respectively.

### Sensitivity analyses

The differences in costs and health benefits among the three treatments in each simulation were visually presented in a cost-effectiveness plane and via the cost-effectiveness acceptability curve. When considering individuals starting from an asymptomatic, cardiovascular disease-free, and recently diagnosed T2DM at baseline, the analysis indicated that pioglitazone was less effective in maximizing DALYs compared to the SGLT2i and GLP-1A classes, but the costs of the treatments varied significantly (Table 2).

The probabilistic sensitivity analyses for the primary care scenario are depicted in Fig. 3b. The cost-effectiveness plane consistently shows pioglitazone closer to or below any mWTPT. For an mWTPT of 8,900 Int$/DALY (equivalent to the Brazilian per capita GDP), pioglitazone emerges as the alternative with the highest probability of being cost-effective (Fig. 3c). One-way sensitivity analyses are illustrated in Supplemental Figures S6a to S6d and consistently indicate pioglitazone as having the highest probability of being cost-effective compared to SGLT2i and GLP-1A.

In the secondary prevention context, probabilistic sensitivity analyses (Fig. 4b) conducted with 5% discount rates for effectiveness and costs show that all drugs are well above the mWTPT of 8,900 Int$/DALY. However, in the acceptability curves (Fig. 4c), pioglitazone exhibited the highest probability of being cost-effective when compared to SGLT2i and GLP-1A. These findings were further confirmed in one-way sensitivity analyses (Supplemental Figures S6e to S6h).

## Discussion

In this study, we confirmed through a systematic quantitative review that pioglitazone, SGLT2i, and GLP-1A are associated with reduced risk of MACE. We also confirmed that SGLT2i and GLP-1A reduced cardiovascular mortality and that pioglitazone increased the incidence of HHF. These data were applied to a cost-effectiveness analysis by BN modeling considering the entire life cycle of individuals in two large real-world T2DM cohorts based in Brazil. Our main finding was that there is a higher probability of increased cost-effectiveness with pioglitazone followed by SGLT2i and GLP-1A, respectively. Furthermore, we found that both SGLT2i and pioglitazone had a high chance of being cost-effective in both primary and secondary prevention scenarios using estimated treatment costs in Brazil, whereas GLP-1A would have a moderate chance of being cost-effective among individuals with T2DM with established coronary artery disease (CAD) but no chance of being cost-effectiveness in subjects without CAD.

CVD risk remains elevated in T2DM individuals even after optimal control of blood pressure, cholesterol levels, and glycated hemoglobin [22]. This residual risk can be mitigated by selecting cardioprotective drugs such as pioglitazone, SGLT2i, or GLP-1A, which have effects that are additive to glycemic control. This strategy, however, must also be conceived from a cost-effectiveness perspective. The annual hospitalization and medication costs are considered the largest share of the global cost resulting from T2DM [23]. Patients with T2DM are hospitalized more than 3 times more frequently than the general population and at least a quarter of these hospitalizations are due to cardiovascular events [24, 25]. The affordability of medicines is a growing challenge for healthcare systems around the world and it is particularly puzzling in low- to middle-income countries where medicines represent 25 to 66% of total public and private spending on health and is the largest domestic expenditure after food [26–28]. In Brazil, for example, current estimates support that the total economic burden of T2DM is US$15.67 billion, which answers for 0.52% of the gross domestic product and up to 5.9% of the total public health expenditures [29]; 44% of this spending is on direct costs [29]. Thus, it is essential to design hierarchical therapeutic protocols that can minimize the escalation of expenses and at the same time reduce the number and severity of high-cost complications.

### Limitations

Some aspects of this study must be considered with some caution. These analyses are based on the average price data of therapies in Brazil, which limits extrapolation of findings to other low to medium-income countries given that drug prices can vary significantly. Furthermore, incidence rates of several outcomes are based on small cohorts, leading to uncertainty regarding statistical power to detect less frequent clinical outcomes. On the other hand, theses cohorts indeed increase internal validity of cost-effectiveness analyses for Brazilian population.

Estimates of the effect size of each therapy were calculated from randomized trials that may exaggerate real-world long-term effectiveness. In another broad meta-analysis [5], the hazard ratios for MACE were significantly different from our findings; and we believe these differences were mainly because we used a network meta-analysis framework, which also considers indirect effect, and the comparator to therapies in the present meta-analysis were always sulfonylureas while Zhu et al [5] had mixed comparators. Although commonly used, the cost–benefit threshold we used in this assessment (1 × GDP per capita) is not consensual and is subject to variation over time, whether due to the loss of patents on therapies or a change in the willingness of payers to pay for them. In this context, a reduction of 71% in the average cost of SGLT2i and of 93% in the average cost of GLP-1A would make them as cost-effective as pioglitazone in a primary prevention scenario. Significant price drops are expected in less than 5 years from now as several new SGLT2i and GLP-1A are available, and patents may expire soon [30].

Consistent with other meta-analyses and the threefold lower incidence rates of HF compared to MACE, our results suggest that the 44% increased relative risk for HHF with pioglitazone may be outweighed by the 27% decrease in the relative risk of MACE [31, 32]. Despite this favorable balance, it is essential to consider that protocols with pioglitazone presuppose the identification and exclusion of patients at greater risk of manifesting heart failure. Since we had no data on endpoints relevant to kidney outcomes or liver cirrhosis due to steatohepatitis, we were unable to estimate the effect of SGLT2i, GLP-1A, or pioglitazone therapies on the costs related to these outcomes. However, the incidence rates of end-stage renal disease from diabetic nephropathy ranged from 38.4 to 804.0 per 100,000 person-years, and liver cirrhosis is found in about 0.2% of the T2DM population [33, 34]. Thus, impacts on the cost-effectiveness of these drugs in the general population of individuals with T2DM would be more salient in cohorts with larger sizes and longer follow-ups. Finally, our model did not take into account the effects of body weight change on non-cardiovascular health, which could in the long-term bolster the cost-effectiveness of GLP-1A.

## Conclusions

In summary, our study reveals a higher likelihood of improved cost-effectiveness with pioglitazone, followed by SGLT2i, and finally GLP-1A for individuals with T2DM in Brazil. These findings underscore the importance of incorporating cost-effectiveness considerations when developing an optimized and hierarchical therapeutic approach for managing T2DM in similar clinical contexts.

### Supplementary Information

**Additional file 1: Supplementary Table S1.** Data Source and Search. **Supplementary Table S2.** Baseline characteristics of patients in the 157 randomized controlled trials (176 study arms). **Supplementary Table S3.** Risk of bias analysis. **Supplementary Table S4.** Egger's Regression Tests for Funnel Plot Asymmetry. **Supplementary Table S5.** Baseline data of participants in Brazilian Diabetes Study cohort (outpatient diabetic individuals). **Supplementary Table S6.** Baseline data of diabetic participants in B-CaRe:QCOR cohort (acute coronary syndromes registry). **Supplementary Figure S1.** Study network. **Supplementary Figure S2.** Evaluation of publication bias in funnel plots for (a) Non-fatal MACE; (b) All-cause deaths; (c) Hospitalization due to heart failure. **Supplementary Figure S3.** Forest plot comparing antidiabetic therapies for the occurrence of non-fatal major cardiovascular adverse events (MACE) in a (a) non-additive and (b) additive effects network meta-analysis with a random-effects model. **Supplementary Figure S4.** Forest plot comparing antidiabetic therapies for the occurrence of all-cause death in a (a) non-additive and (b) additive effects network meta-analysis with a random-effects model. **Supplementary Figure S5.** Forest plot comparing antidiabetic therapies for hospitalizations due to heart failure (HHF) in a (a) non-additive and (b) additive effects network meta-analysis with a random-effects model. **Supplementary Figure S6.** One-way sensitivity analyses for (a to d) the scenario where individual depart from state A (asymptomatic, primary prevention) and for (e to h) the scenario where individual depart from state C (recent acute coronary syndrome or stroke, secondary prevention).

## Data Availability

Not applicable.

## Abbreviations

T2DM: Type 2 Diabetes Mellitus
CVD: Cardiovascular disease
SGLT2i: Sodium-glucose transporter-2 inhibitors
GLP-1A: Glucagon-like peptide 1 receptor agonists
MACE: Major cardiovascular events
BN: Bayesian network
MID: Markov influence diagram
PRISMA: Systematic Reviews and Meta-Analyzes
PubMed: Electronic databases Medline
WHO: World Health Organization
RCT: Randomized clinical trials
HHF: Hospitalization for heart failure
BMI: Body mass index
HbA1c: Hemoglobin A1c
eGFR: Estimated glomerular filtration rate
CNMA: Network meta-analysis
HRs: Hazard ratios
BDS: Brazilian Diabetes Study
B-CaReQCO: Brasilia Cardiovascular Registry for Quality of Care and Outcomes
SUS: Brazilian Unified Health System
R$: Reais
Int$: International dollars
PPP: Purchasing power parity
OPS: Out-of-pocket spending
IPCA: National Wide Consumer Price Index
IBGE: Brazilian Institute of Geography and Statistics
ICER: Incremental cost-effectiveness ratio
DALYs: Disease-adjusted life years
GDP: Per capita gross domestic product
mWTPT: Maximum willingness to pay threshold
CAD: Coronary artery disease

## References

[1] Bommer C, Sagalova V, Heesemann E, Manne-Goehler J, Atun R, Bärnighausen T, Davies J, Vollmer S (2018). Global economic burden of diabetes in adults: projections from 2015 to 2030. Diabetes Care.

[2] Sun H, Saeedi P, Karuranga S, Pinkepank M, Ogurtsova K, Duncan BB, Stein C, Basit A, Chan JCN, Mbanya JC (2022). IDF Diabetes atlas: global, regional and country-level diabetes prevalence estimates for 2021 and projections for 2045. Diabetes Res Clin Pract.

[3] Einarson TR, Acs A, Ludwig C, Panton UH (2018). Prevalence of cardiovascular disease in type 2 diabetes: a systematic literature review of scientific evidence from across the world in 2007–2017. Cardiovasc Diabetol.

[4] Einarson TR, Acs A, Ludwig C, Panton UH (2018). Economic burden of cardiovascular disease in type 2 diabetes: a systematic review value. Health.

[5] Zhu J, Yu X, Zheng Y, Li J, Wang Y, Lin Y, He Z, Zhao W, Chen C, Qiu K (2020). Association of glucose-lowering medications with cardiovascular outcomes: an umbrella review and evidence map The lancet. Diabetes Endocrinol.

[6] Arora P, Boyne D, Slater JJ, Gupta A, Brenner DR, Druzdzel MJ (2019). Bayesian networks for risk prediction using real-world data: a tool for precision medicine. Value Health.

[7] Shafie AA, Tan YP, Ng CH (2017). Systematic review of economic burden of heart failure. Heart Failure Rev.

[8] Bahia LR, da Machado Rosa MQ, Araujo DV, Correia MG, Dos Santos Dos RR, Duncan BB, Toscano CM (2019). Economic burden of diabetes in Brazil in 2014. Diabetol Metabol Syndr.

[9] Stratton IM, Adler AI, Neil HA, Matthews DR, Manley SE, Cull CA, Hadden D, Turner RC, Holman RR (2000). Association of glycaemia with macrovascular and microvascular complications of type 2 diabetes (UKPDS 35): prospective observational study. BMJ (Clinical Research ed.).

[10] Iribarren C, Karter AJ, Go AS, Ferrara A, Liu JY, Sidney S, Selby JV (2001). Glycemic control and heart failure among adult patients with diabetes. Circulation.

[11] Margulies KB, Hernandez AF, Redfield MM, Givertz MM, Oliveira GH, Cole R, Mann DL, Whellan DJ, Kiernan MS, Felker GM (2016). Effects of Liraglutide on Clinical stability among patients with advanced heart failure and reduced ejection fraction: a randomized clinical trial. JAMA.

[12] Vaduganathan M, Docherty KF, Claggett BL, Jhund PS, de Boer RA, Hernandez AF, Inzucchi SE, Kosiborod MN, Lam CSP, Martinez F (2022). SGLT-2 inhibitors in patients with heart failure: a comprehensive meta-analysis of five randomised controlled trials. Lancet (London, England).

[13] Solomon SD, McMurray JJV, Claggett B, de Boer RA, DeMets D, Hernandez AF, Inzucchi SE, Kosiborod MN, Lam CSP, Martinez F (2022). Dapagliflozin in heart failure with mildly reduced or preserved ejection fraction. N Engl J Med.

[14] Anker SD, Butler J, Filippatos G, Ferreira JP, Bocchi E, Böhm M, Rocca HPBL, Choi DJ, Chopra V, Chuquiure-Valenzuela E (2021). Empagliflozin in heart failure with a preserved ejection fraction. N Engl J Med.

[15] McMurray JJV, Solomon SD, Inzucchi SE, Køber L, Kosiborod MN, Martinez FA, Ponikowski P, Sabatine MS, Anand IS, Bělohlávek J (2019). Dapagliflozin in patients with heart failure and reduced ejection fraction. New Engl J Med.

[16] Packer M, Anker SD, Butler J, Filippatos G, Pocock SJ, Carson P, Januzzi J, Verma S, Tsutsui H, Brueckmann M (2020). Cardiovascular and renal outcomes with Empagliflozin in heart failure. N Engl J Med.

[17] Bhatt DL, Szarek M, Steg PG, Cannon CP, Leiter LA, McGuire DK, Lewis JB, Riddle MC, Voors AA, Metra M (2021). Sotagliflozin in patients with diabetes and recent worsening heart failure. N Engl J Med.

[18] Sonnenberg FA, Beck JR (1993). Markov models in medical decision making: a practical guide. Med Decis Making.

[19] de Menezes FG, Barreto DV, Abreu RM, Roveda F, Filho RFSP (2015). Overview of hemodialysis treatment funded by the Brazilian unified health system–an economic perspective. J Bras Nefrol.

[20] Marseille E, Larson B, Kazi DS, Kahn JG, Rosen S (2015). Thresholds for the cost-effectiveness of interventions: alternative approaches. Bull World Health Org.

[21] Brasil. Ministério da saúde. Secretaria de Ciência, T. e. I. E. D. d. C. e. T. Diretrizes metodológicas. Diretriz de Avaliação Econômica. 2ª edição.; Ministério da Saúde, 2014. ISBN 978-85-334-2182-0.

[22] Lin FJ, Tseng WK, Yin WH, Yeh HI, Chen JW, Wu CC (2017). Residual risk factors to predict major adverse cardiovascular events in atherosclerotic cardiovascular disease patients with and without diabetes mellitus. Scient Rep.

[23] Moucheraud C, Lenz C, Latkovic M, Wirtz VJ (2019). The costs of diabetes treatment in low- and middle-income countries: a systematic review. BMJ Global Health.

[24] Moss SE, Klein R, Klein BE (1999). Risk factors for hospitalization in people with diabetes. Arch Int Med.

[25] Donnan PT, Leese GP, Morris AD (2000). Hospitalizations for people with type 1 and type 2 diabetes compared with the nondiabetic population of Tayside, Scotland: a retrospective cohort study of resource use. Diabetes Care.

[26] Silveira FG, Osório RG, Piola SF (2023). Os gastos das famílias com saúde. Ciência Saúde Coletiva.

[27] WHO. Medicines Pricing and Financing. 2018. [cited 2018 July 13th] Available from: http://www.who.int/medicines/areas/access/en/.

[28] WHO. Essential medicines. 2018. [cited 2018 July 16th] Available from: http://www.who.int/medicines/services/essmedicines_def/en/.

[29] Bahia LR, Araujo DV, Schaan BD, Dib SA, Negrato CA, Leão MP, Ramos AJ, Forti AC, Gomes MB, Foss MC (2011). The costs of type 2 diabetes mellitus outpatient care in the Brazilian public health system. Value Health.

[30] Taylor SI (2020). The high cost of diabetes drugs: disparate impact on the most vulnerable patients. Diabetes Care.

[31] Varas-Lorenzo C, Margulis AV, Pladevall M, Riera-Guardia N, Calingaert B, Hazell L, Romio S, Perez-Gutthann S (2014). The risk of heart failure associated with the use of noninsulin blood glucose-lowering drugs: systematic review and meta-analysis of published observational studies. BMC Cardiovasc Disord.

[32] Lincoff AM, Wolski K, Nicholls SJ, Nissen SE (2007). Pioglitazone and risk of cardiovascular events in patients with type 2 diabetes mellitus: a meta-analysis of randomized trials. JAMA.

[33] Narres M, Claessen H, Droste S, Kvitkina T, Koch M, Kuss O, Icks A (2016). The incidence of end-stage renal disease in the diabetic (Compared to the Non-Diabetic) population: a systematic review. PloS One.

[34] Williamson RM, Price JF, Hayes PC, Glancy S, Frier BM, Johnston GI, Reynolds RM, Strachan MW (2012). Prevalence and markers of advanced liver disease in type 2 diabetes. QJM : Monthly J Assoc Phys.

