# Supplementary material for: Comparative effectiveness and cost-effectiveness of cardioprotective glucose-lowering therapies for type 2 diabetes in Brazil: a Bayesian network model
Source: Health Econ Rev. 2023 Oct 25;13:50. doi: 10.1186/s13561-023-00466-3 (PMC10599033; doi:10.1186/s13561-023-00466-3)
Supplement: Supplementary file 1 — Additional file 1: Supplementary Table S1. Data Source and Search. Supplementary Table S2. Baseline characteristics of patients in the 157 randomized controlled trials (176 study arms). Supplementary Table S3. Risk of bias analysis. Supplementary Table S4. Egger's Regression Tests for Funnel Plot Asymmetry. Supplementary Table S5. Baseline data of participants in Brazilian Diabetes Study cohort (outpatient diabetic individuals). Supplementary Table S6. Baseline data of diabetic participants in B-CaRe:QCOR cohort (acute coronary syndromes registry). Supplementary Figure S1. Study network. Supplementary Figure S2. Evaluation of publication bias in funnel plots for (a) Non-fatal MACE; (b) All-cause deaths; (c) Hospitalization due to heart failure. Supplementary Figure S3. Forest plot comparing antidiabetic therapies for the occurrence of non-fatal major cardiovascular adverse events (MACE) in a (a) non-additive and (b) additive effects network meta-analysis with a random-effects model. Supplementary Figure S4. Forest plot comparing antidiabetic therapies for the occurrence of all-cause death in a (a) non-additive and (b) additive effects network meta-analysis with a random-effects model. Supplementary Figure S5. Forest plot comparing antidiabetic therapies for hospitalizations due to heart failure (HHF) in a (a) non-additive and (b) additive effects network meta-analysis with a random-effects model. Supplementary Figure S6. One-way sensitivity analyses for (a to d) the scenario where individual depart from state A (asymptomatic, primary prevention) and for (e to h) the scenario where individual depart from state C (recent acute coronary syndrome or stroke, secondary prevention). [file 13561_2023_466_MOESM1_ESM.docx]

**Supplementary material**

**Supplementary Table S1:**  Data Source and Search

| **Items** | **N.** | **Terms** |
| --- | --- | --- |
| Disease | #1 | " type 2 diabetes mellitus"[MeSH Terms] OR ("type 2 diabetes"[All Fields] |
| SGLT2i | #2 | "canagliflozin"[MeSH Terms] OR "canagliflozin"[All Fields] |
|  | #3 | "empagliflozin"[MeSH Terms] OR "empagliflozin"[All Fields] |
|  | #4 | "dapagliflozin"[MeSH Terms] OR "dapagliflozin"[All Fields] |
|  | #5 | “ertugliflozin"[MeSH Terms] OR "ertugliflozin"[All Fields] |
|  | #6 | “ipragliflozin"[MeSH Terms] OR "ipragliflozin"[All Fields] |
|  | #7 | “tofogliflozin"[MeSH Terms] OR "tofogliflozin"[All Fields] |
| GLP-1A | #8 | "lixisenatide"[MeSH Terms] OR "lixisenatide"[All Fields] |
|  | #9 | "liraglutide"[MeSH Terms] OR "liraglutide"[All Fields] |
|  | #10 | "semaglutide"[MeSH Terms] OR "semaglutide"[All Fields] |
|  | #11 | "albiglutide"[All Fields] OR "rGLP-1 protein"[Supplementary Concept] OR "rGLP-1 protein"[All Fields] |
|  | #12 | "exenatide"[MeSH Terms] OR "exenatide"[All Fields] |
|  | #13 | "dulaglutide"[Supplementary Concept] OR "dulaglutide"[All Fields] |
| DPP4i | #14 | "alogliptin"[MeSH Terms] OR "alogliptin"[All Fields] |
|  | #15 | "linagliptin"[MeSH Terms] OR "linagliptin"[All Fields] |
|  | #16 | "omarigliptin"[MeSH Terms] OR "omarigliptin"[All Fields] |
|  | #17 | "saxagliptin"[MeSH Terms] OR "saxagliptin"[All Fields] |
|  | #18 | "sitagliptin"[MeSH Terms] OR "sitagliptin"[All Fields] |
|  | #19 | "vildagliptin"[MeSH Terms] OR "vildagliptin"[All Fields] |
| Sulfonylurea | #20 | "glibenclamide"[MeSH Terms] OR "glibenclamide"[All Fields] |
|  | #21 | "gliclazide"[MeSH Terms] OR “gliclazide"[All Fields] |
|  | #22 | "glimepiride"[MeSH Terms] OR "glimepiride"[All Fields] |
|  | #23 | "glipizide"[MeSH Terms] OR "glipizide"[All Fields] |
| Thiazolidinedione | #24 | "pioglitazone"[MeSH Terms] OR "pioglitazone"[All Fields] |
| Biguanide | #25 | "metformin"[MeSH Terms] OR "metformin"[All Fields] |
| Glinide | #26 | "nateglinide"[MeSH Terms] OR "neteglinide"[All Fields] |
|  | #27 | "repaglinide"[MeSH Terms] OR "repaglinide"[All Fields] |
| Study design | #28 | "randomized controlled trials "[MeSH Terms] OR "randomized controlled trial"[All Fields] OR controlled clinical trial[Publication Type]) |
| Filters | #29 | "humans"[MeSH Terms] |
|  | | |
| **Strings** | | |
| 1^st^ search - SGLT2i | #30 | #1 AND (#2 OR #3 OR #4 OR #5 OR #6 OR #7) AND #28 AND #29 |
| 2^nd^ search - GLP1A | #31 | #1 AND (#8 OR #9 OR #10 OR #11 OR #12 OR #13) AND #28 AND #29 |
| 3^th^ search - DPP4i | #32 | #1 AND (#14 OR #15 OR #16 OR #17 OR #18 OR #19) AND #28 AND #29 |
| 4^th^ search - Sulfonylurea | #33 | #1 AND (#20 OR #21 OR #22 OR #23) AND #28 AND #29 |
| 5^th^ search – Thiazolidinedione | #34 | #1 AND #24 AND #28 AND #29 |
| 6^th^ search – Biguanide | #35 | #1 AND #25 AND #28 AND #29 |
| 7^th^ search – Glinide | #36 | #1 AND (#26 OR #27) AND #28 AND #29 |

Queries of literature were performed using the electronic databases Medline (PubMed), ClinicalTrials.gov, Cochrane Central Register of Controlled Trials, Embase, European Union Clinical Trials Register and World Health Organization (WHO) International Clinical Trials Registry Platform. The search included all submitted articles until 12^st^ of April 2021 with no restriction to submission date or language, however all articles relevant to this study were published in English. The search was filtered to include only randomized controlled trials (RCTs) involving humans. Systematic reviews and meta-analyses were also evaluated to identify other relevant RCTs that were eventually missing by using the search terms

**Supplementary Table S2.** Baseline characteristics of patients in the 157 randomized controlled trials (176 study arms)

| RCT | Active arm | Comparator arm | Follow-up time (years, mean) | Total sample size (n) | Exposure (patient-years) | Whites (%, mean) | Women (%, mean) | Age (years, mean±SD) | | | Time since DM diagnosis (years) | | | HbA1c at baseline (%, mean±SD) | | |
| --- | --- | --- | --- | --- | --- | --- | --- | --- | --- | --- | --- | --- | --- | --- | --- | --- |
|  |  |  |  |  |  |  |  |  |  |  |  |  |  |  |  |  |
| AWARD-3 (1) | GLP1A | Biguanide | 0,96 | 807 | 775 | 75,0 | 55,0 | 55,00 | ± | 10,00 | 3,00 | ± | 2,00 | 7,60 | ± | 0,80 |
| AWARD-5 (2) | GLP1A | DPP4i | 1,90 | 921 | 1750 | 50,0 | 52,0 | 54,00 | ± | 10,00 | 7,00 | ± | 5,00 | 8,10 | ± | 1,10 |
| AWARD-9 (3) | GLP1A | Placebo | 0,53 | 300 | 159 | 92,0 | 41,3 | 60,60 | ± | 10,10 | 13,30 | ± | 7,70 | 8,30 | ± | 0,80 |
| AWARD-10 (4) | GLP1A | Placebo | 0,46 | 423 | 195 | 89,0 | 53,0 | 57,10 | ± | 9,59 | 8,87 | ± | 6,13 | 8,05 | ± | 0,66 |
| Ahmann (2015) (5) | GLP1A | Placebo | 0,5 | 450 | 225 | 39,6 | 39,6 | 57,50 | ± | 11,10 | 12,10 | ± | 6,80 | 8,30 | ± | 0,90 |
| Arechavaleta (2010) (6) | DPP4i | Sulfonylurea | 0,58 | 1035 | 600 | 57,4 | 53,8 | 56,20 | ± | 10,10 | 6,70 | ± | 4,80 | 7,50 | ± | 0,80 |
| Arjona (2013) (7) | DPP4i | Sulfonylurea | 1,00 | 423 | 423 | 28,2 | 45,1 | 64,30 | ± | 9,20 | 10,10 | ± | 7,80 | 7,80 | ± | 0,70 |
| Aroda (2016) (8) | GLP1A+Insulin | GLP1A+Placebo | 0,50 | 356 | 178 | 87,8 | 39,5 | 57,30 | ± | 9,40 | 9,30 | ± | 5,40 | 7,60 | ± | 0,60 |
| Bailey (2013) (9) | SGLT2i | Placebo | 2,00 | 546 | 1092 |  | 45,0 | 53,70 | ± | 10,30 | 5,80 | ± | 5,10 | 8,11 | ± | 0,96 |
| Bailey (2015) (10) | SGLT2i | Placebo | 2,00 | 274 | 548 | 94,7 | 58,7 | 52,70 | ± | 10,30 | 2,10 | ± | 3,10 | 7,84 | ± | 0,87 |
| Barnett (2013) (11) | DPP4i | Placebo | 0,96 | 455 | 437 | 78,1 | 55,0 | 57,30 | ± | 9,30 | 12,20 | ± | 7,40 | 8,60 | ± | 0,90 |
| Barnett (2014) (12) | SGLT2i | Placebo | 1,00 | 374 | 374 | 57,8 | 43,3 | 65,10 | ± | 8,20 |  |  |  | 8,09 | ± | 0,80 |
| Bode (2015) (13) | SGLT2i | Placebo | 1,96 | 714 | 1399 | 78,1 | 39,7 | 63,20 | ± | 6,20 | 11,40 | ± | 7,30 | 7,80 | ± | 0,80 |
| Bolli (2007) (14) | TZD | DPP4i | 0,44 | 576 | 253 | 82,4 | 38,3 | 56,30 | ± | 9,30 | 6,40 | ± | 4,90 | 8,40 | ± | 1,00 |
| Bolli (2009) (15) | DPP4i | SGLT2i | 0,96 | 576 | 553 | 81,9 | 35,9 | 57,00 | ± | 9,70 | 6,40 | ± | 4,90 | 8,40 | ± | 0,90 |
| Bosi (2007) (16) | DPP4i | Placebo | 0,46 | 544 | 250 | 73,1 | 46,9 | 54,50 | ± | 10,30 | 6,20 | ± | 5,30 | 8,30 | ± | 0,90 |
| Bosi (2011) (17) | DPP4i | SGLT2i | 0,96 | 803 | 771 | 64,2 | 48,9 | 55,90 | ± | 9,94 | 6,90 | ± | 4,61 | 8,10 | ± | 0,83 |
| Buse (2011) (18) | GLP1A+Insulin | Insulin+Placebo | 0,57 | 259 | 148 | 80,0 | 36,0 | 59,00 | ± | 10,00 | 12,00 | ± | 7,00 | 8,50 | ± | 0,96 |
| CANVAS (19) | SGLT2i | Placebo | 3,50 | 10142 | 35497 | 79,0 | 36,7 | 63,40 | ± | 8,20 | 13,70 | ± | 7,80 | 8,20 | ± | 0,90 |
| CARMELINA (20) | DPP4i | Placebo | 2,20 | 6979 | 15354 | 79,5 | 35,7 | 65,60 | ± | 9,10 | 14,50 | ± | 9,30 | 8,00 | ± | 1,00 |
| CAROLINA (21) | DPP4i | Sulfonylurea | 6,30 | 6033 | 38008 | 73,0 | 40,8 | 64,20 | ± | 9,50 | 6,20 |  |  | 7,20 | ± | 0,60 |
| Cannon (2020) (22) | SGLT2i | Placebo | 3,50 | 8246 | 28861 | 87,8 | 29,7 | 64,40 | ± | 8,10 | 12,90 | ± | 8,30 | 8,20 | ± | 1,00 |
| Cefalu (2015) (23) | SGLT2i | Placebo | 0,96 | 914 | 877 | 85,2 | 31,4 | 63,00 | ± | 7,70 | 12,30 | ± | 8,20 | 8,08 | ± | 0,80 |
| Chacra (2011) (24) | DPP4i | Placebo | 1,40 | 769 | 1077 | 56,9 | 53,9 | 55,10 | ± | 10,70 | 6,80 | ± | 5,70 | 8,40 | ± | 0,90 |
| Charbonnel (2005)-1 (25) | TZD+Sulfonylurea | Biguanide+Sulfonylurea | 2,00 | 639 | 1278 | 98,4 | 45,3 | 60,00 |  | 8,00 | 7,10 |  | 5,60 | 8,80 |  | 0,97 |
| Charbonnel (2005)-2 (25) | TZD+Biguanide | Biguanide+Sulfonylurea | 2,00 | 630 | 1269 | 100,0 | 50,8 | 57,00 |  | 9,00 | 5,50 |  | 5,10 | 8,52 |  | 0,90 |
| Chen (2018) (26) | GLP1A | Sulfonylurea | 0,50 | 720 | 360 |  | 46,3 | 52,00 | ± | 10,05 | 3,80 | ± | 4,09 | 7,90 | ± | 1,01 |
| DECLARE (27) | SGLT2i | Placebo | 4,20 | 17160 | 72072 | 79,4 | 37,9 | 64,00 | ± | 6,80 | 10,00 |  |  | 8,30 | ± | 1,20 |
| DUAL IV (28) | GLP1A+Insulin | Placebo | 0,50 | 435 | 218 | 76,0 | 50,0 | 59,40 | ± | 10,80 | 9,30 | ± | 6,50 | 7,90 | ± | 0,60 |
| DURATION-2 (1) (29) | GLP1A | DPP4i | 0,50 | 326 | 163 | 30,0 | 48,0 | 52,00 | ± | 11,00 | 5,00 | ± | 4,00 | 8,50 | ± | 1,20 |
| DURATION-2 (2) (29) | GLP1A | TZD | 0,50 | 325 | 166 | 39,0 | 52,0 | 53,00 | ± | 10,00 | 6,00 | ± | 4,00 | 8,50 | ± | 1,10 |
| DURATION-4 (1) (30) | GLP1A | Biguanide | 0,50 | 494 | 205 | 65,0 | 37,4 | 54,00 | ± | 11,00 | 2,60 | ± | 3,60 | 8,60 | ± | 1,20 |
| DURATION-4 (2) (30) | GLP1A | TZD | 0,50 | 411 | 206 | 67,5 | 40,5 | 55,00 |  | 11,00 | 2,70 | ± | 3,70 | 8,50 | ± | 1,20 |
| DURATION-7 (31) | GLP1A+Insulin | Insulin+Placebo | 0,53 | 461 | 244 | 85,2 | 53,5 | 57,60 | ± | 10,30 | 11,10 | ± | 6,10 | 8,53 | ± | 0,92 |
| DURATION-8 (32) | GLP1A | SGLT2i | 0,96 | 457 | 439 | 82,2 | 52,2 | 54,50 | ± | 9,20 | 7,10 | ± | 5,50 | 9,30 | ± | 1,00 |
| DURATION-8 (32) | GLP1A+SGLT2i | SGLT2i | 0,96 | 458 | 440 | 82,2 | 52,2 | 54,50 | ± | 9,20 | 7,10 | ± | 5,50 | 9,30 | ± | 1,00 |
| DURATION-8 (32) | GLP1A+SGLT2i | GLP1A | 0,96 | 455 | 437 | 85,5 | 48,9 | 54,20 | ± | 9,60 | 7,40 | ± | 5,50 | 9,30 | ± | 1,10 |
| DeFronzo (2009) (33) | DPP4i | Placebo | 0,46 | 743 | 342 | 83,8 | 46,4 | 54,80 | ± | 10,20 | 6,70 | ± | 5,60 | 8,10 | ± | 0,90 |
| DeFronzo (2012) (34) | TZD | Placebo | 0,48 | 518 | 249 | 68,6 | 54,4 | 54,90 | ± | 9,50 | 6,60 | ± | 5,40 | 8,50 | ± | 0,70 |
| DeFronzo (2015)-1 (35) | SGLT2i+DPP4i | DPP4i | 1,00 | 397 | 397 | 75,0 | 50,0 | 56,20 |  | 10,00 |  |  |  | 8,02 |  | 0,90 |
| DeFronzo (2015)-2 (35) | SGLT2i | DPP4i | 1,00 | 405 | 495 | 75,0 | 50,0 | 56,20 |  | 10,00 |  |  |  | 8,02 |  | 0,90 |
| Del Prato (2014) (36) | DPP4i | Sulfonylurea | 1,92 | 2639 | 5067 | 61,0 | 49,5 | 55,40 | ± | 9,60 | 5,50 | ± | 4,88 | 7,60 | ± | 0,62 |
| Del Prato (2015) (37) | SGLT2i | Sulfonylurea | 4,00 | 801 | 3204 |  | 45,1 | 58,60 | ± | 9,80 | 6,60 | ± | 5,90 | 7,74 | ± | 0,89 |
| Derumeaux (2012) (38) | Benfluorex | TZD | 1,00 | 846 | 846 | 75,0 | 47,0 | 59,60 | ± | 10,30 | 7,40 | ± | 5,95 | 8,30 | ± | 0,80 |
| Dormandy (2005) (39) | TZD | Placebo | 2,88 | 5238 | 15059 | 99,0 | 34,0 | 61,60 | ± | 7,80 | 8,00 |  |  | 7,90 |  |  |
| ELIXA (40) | GLP1A | Placebo | 2,08 | 6068 | 12621 | 76,4 | 30,9 | 60,60 | ± | 9,60 | 9,40 | ± | 8,30 | 7,60 | ± | 1,30 |
| EMPA-REG (41) | SGLT2i | Placebo | 3,10 | 7020 | 21762 | 71,9 | 28,0 | 63,20 | ± | 8,80 |  |  |  | 8,08 | ± | 0,80 |
| EXAMINE (42) | DPP4i | Placebo | 1,50 | 5380 | 8070 | 72,2 | 32,0 | 60,70 | ± | 9,88 | 9,18 | ± | 8,12 | 8,00 | ± | 1,10 |
| EXSCEL (43) | GLP1A | Placebo | 3,20 | 14752 | 47206 | 76,0 | 38,0 | 62,00 | ± | 8,70 | 12,00 | ± | 8,30 | 8,00 | ± | 0,97 |
| FREEDOM-1 (44) | GLP1A | Placebo | 0,74 | 460 | 340 | 81,8 | 40,30 | 54,70 |  | 9,10 | 8,60 |  | 6,00 | 8,50 |  | 0,80 |
| Ferdinand (2014) (45) | GLP1A | Placebo | 0,50 | 755 | 378 | 81,2 | 47,6 | 56,40 | ± | 10,50 | 8,40 | ± | 5,80 | 7,90 | ± | 0,80 |
| Ferrannini (2009) (46) | Sulfonylurea | DPP4i | 1,00 | 2789 | 2789 | 86,3 | 47,2 |  |  |  | 5,71 | ± | 5,18 | 7,31 | ± | 0,64 |
| Filozof (2010) (47) | DPP4i | Sulfonylurea | 0,96 | 1007 | 967 | 77,5 | 48,2 | 59,70 |  | 10,20 | 6,80 |  | 5,30 | 8,50 |  | 1,00 |
| Foley (2009) (48) | DPP4i | Sulfonylurea | 2,00 | 1092 | 2184 | 73,40 | 47,3 | 54,30 |  | 10,40 | 1,20 |  | 3,10 | 8,70 |  | 1,10 |
| Frias (2020) (49) | SGLT2i+DPP4i | Sulfonylurea | 1,00 | 443 | 433 | 90,3 | 53,2 | 56,10 | ± | 9,20 | 7,90 | ± | 6,50 | 8,50 | ± | 0,80 |
| Gallwitz (2012) (50) | DPP4i | Sulfonylurea | 2,00 | 1551 | 3102 | 85,0 | 39,0 | 59,80 | ± | 9,40 |  |  |  | 7,70 | ± | 0,90 |
| Gantz (2017) (51) | DPP4i | Placebo | 1,80 | 4202 | 7564 | 81,3 | 29,3 | 63,60 | ± | 8,50 | 12,10 | ± | 8,00 | 8,00 | ± | 0,90 |
| Gerich (2005) (52) | Glinide+Biguanide | Biguanide+Sulfonylurea | 2,00 | 428 | 856 | 65,2 | 52,0 | 53,50 |  | 11,60 | 2,00 |  | 4,30 | 8,30 |  | 1,10 |
| GetGoal (53) | GLP1A | Placebo | 1,40 | 482 | 675 | 93,0 | 55,0 | 58,20 | ± | 9,80 | 6,20 | ± | 4,70 | 8,00 | ± | 0,80 |
| GetGoal-L (54) | GLP1A | Placebo | 0,50 | 495 | 248 | 78,0 | 51,0 | 57,00 | ± | 10,00 | 12,40 | ± | 7,00 | 8,40 | ± | 0,80 |
| GetGoal-L-Asia (55) | GLP1A | Placebo | 0,46 | 311 | 143 |  | 49,0 | 58,00 | ± | 10,10 | 14,10 | ± | 7,70 | 8,52 | ± | 0,78 |
| GetGoal-M-Asia (56) | GLP1A | Placebo | 0,46 | 390 | 179 |  | 53,1 | 55,10 | ± | 10,50 | 6,80 | ± | 4,80 | 7,85 | ± | 0,71 |
| GetGoal-O (57) | GLP1A | Placebo | 0,46 | 350 | 161 | 70,1 | 48,3 | 74,40 | ± | 3,80 | 14,60 | ± | 13,60 | 8,05 | ± | 0,70 |
| GetGoal-S (58) | GLP1A | Placebo | 0,46 | 859 | 395 | 53,0 | 47,4 | 57,80 | ± | 10,10 | 9,80 | ± | 6,20 | 8,20 | ± | 0,80 |
| Giles (2008) (59) | TZD | Sulfonylurea | 0,50 | 518 | 258 |  | 23,0 | 63,40 | ± | 9,38 | 11,71 | ± | 9,48 | 8,95 |  |  |
| Giles (2010) (60) | TZD | Sulfonylurea | 1,00 | 300 | 300 | 61,0 | 44,0 | 64,00 | ± | 9,90 |  |  |  | 8,30 | ± | 1,10 |
| Goke (2008) (61) | DPP4i | Biguanide | 2,00 | 463 | 926 | 76,6 | 39,0 | 54,08 | ± | 9,64 | 2,20 | ± | 2,89 | 8,80 | ± | 0,10 |
| Goke (2013) (62) | DPP4i | Sulfonylurea | 1,90 | 858 | 1630 | 84,2 | 46,0 | 57,60 | ± | 7,35 | 5,40 | ± | 2,98 | 7,65 | ± | 0,04 |
| Goldstein (2007)-2 (63) | DPP4i | Placebo | 0,46 | 355 | 163 | 46,0 | 48,0 | 53,30 |  | 10,20 | 4,40 |  | 4,60 | 8,87 |  | 0,99 |
| HARMONY (64) | GLP1A | Placebo | 1,50 | 9463 | 14195 | 69,0 | 31,0 | 64,20 | ± | 8,70 | 14,20 | ± | 8,90 | 8,72 | ± | 1,50 |
| HARMONY-1 (65) | GLP1A | Placebo | 2,90 | 301 | 873 | 71,5 | 41,7 | 54,90 | ± | 9,40 | 7,90 | ± | 6,10 | 8,10 | ± | 0,90 |
| HARMONY-3 (1) (66) | GLP1A | Sulfonylurea | 1,90 | 609 | 1157 | 71,7 | 48,5 | 54,40 | ± | 10,00 | 6,00 | ± | 4,80 | 8,10 | ± | 0,80 |
| HARMONY-3 (2) (66) | GLP1A | DPP4i | 1,90 | 604 | 1148 | 74,5 | 54,0 | 54,30 | ± | 9,80 | 5,80 | ± | 4,80 | 8,10 | ± | 0,80 |
| HARMONY-3 (3) (66) | GLP1A | Placebo | 1,90 | 403 | 766 | 63,4 | 50,5 | 56,10 | ± | 10,00 | 6,70 | ± | 6,60 | 8,20 | ± | 0,90 |
| HARMONY-5 (1) (67) | GLP1A | TZD | 2,90 | 548 | 1589 | 73,3 | 46,6 | 55,70 | ± | 9,40 | 9,20 | ± | 6,10 | 8,29 | ± | 0,88 |
| HARMONY-5 (2) (67) | GLP1A | Placebo | 2,90 | 386 | 1119 | 69,6 | 39,1 | 55,70 | ± | 9,60 | 9,30 | ± | 6,10 | 8,26 | ± | 0,98 |
| HARMONY-8 (68) | GLP1A | DPP4i | 0,96 | 495 | 475 | 46,3 | 47,2 | 63,50 | ± | 9,02 | 11,62 | ± | 8,47 | 8,23 | ± | 0,94 |
| Haering (2015) (69) | SGLT2i | Placebo | 1,40 | 666 | 932 | 39,1 | 50,2 | 56,90 | ± | 9,20 |  |  |  | 8,20 | ± | 0,80 |
| Halvorsen (2019) (70) | DPP4i | SGLT2i | 0,44 | 384 | 169 | 82,7 | 37,2 | 59,30 |  | 9,70 | 8,22 |  | 5,70 | 7,94 |  | 0,81 |
| Handelsman (2017) (71) | DPP4i | Sulfonylurea | 1,00 | 751 | 751 | 82,7 | 43,7 | 58,00 | ± | 9,00 | 7,70 | ± | 4,90 | 7,40 | ± | 0,70 |
| Henry (2013)-1 (72) | DPP4i | TZD | 1,04 | 751 | 781 | 67,6 | 41,6 | 51,15 | ± | 9,20 | 3,76 | ± | 4,06 | 8,80 | ± | 1,10 |
| Henry (2013)-2 (72) | DPP4i | DPP4i+TZD | 1,04 | 767 | 798 | 66,6 | 41,6 | 53,40 | ± | 9,10 | 3,97 | ± | 4,13 | 8,80 | ± | 1,10 |
| Henry (2013)-3 (72) | DPP4i+TZD | TZD | 1,04 | 1146 | 1192 | 67,4 | 43,5 | 51,15 | ± | 9,20 | 3,76 | ± | 4,06 | 8,80 | ± | 1,10 |
| Hollander (2011) (73) | DPP4i | Placebo | 1,40 | 565 | 791 | 54,9 | 53,8 | 54,00 | ± | 10,10 | 5,10 | ± | 5,40 | 8,20 | ± | 1,10 |
| Hollander (2019) (74) | SGLT2i | Sulfonylurea | 2,00 | 1305 | 2610 | 72,9 | 48,5 | 57,90 | ± | 9,10 | 7,60 | ± | 5,60 | 7,80 | ± | 0,60 |
| Hong (2013) (75) | Sulfonylurea | Biguanide | 3,00 | 304 | 912 | 0,0 | 21,8 | 62,80 | ± | 8,50 | 5,60 | ± | 5,30 | 7,60 | ± | 1,70 |
| Horton (2000) (76) | Biguanide | Glinide | 0,44 | 357 | 157 | 82,1 | 38,5 | 58,60 |  | 10,70 | 4,70 | ± | 5,50 | 8,30 | ± | 1,00 |
| IDegLira (77) | GLP1A+Insulin | Insulin | 0,50 | 398 | 199 | 76,0 | 47,0 | 58,00 | ± | 11,00 | 11,00 | ± | 7,00 | 8,80 | ± | 0,70 |
| Jabbour (2014) (78) | SGLT2i | Placebo | 0,90 | 447 | 402 | 76,3 | 47,3 | 54,80 |  | 10,40 | 5,64 |  | 5,40 | 8,00 |  | 0,80 |
| Jain (2006) (79) | TZD | Sulfonylurea | 1,00 | 502 | 502 | 65,7 | 45,4 | 52,10 | ± | 12,39 | 9,40 | ± | 15,18 | 9,20 | ± | 1,26 |
| Kawamori (2018) (80) | SGLT2i | Placebo | 0,96 | 275 | 264 |  | 22,6 | 59,80 | ± | 10,80 | 8,70 | ± | 6,10 | 8,36 | ± | 0,74 |
| Kohan (2014) (81) | SGLT2i | Placebo | 1,96 | 252 | 494 | 82,1 | 36,9 | 67,00 | ± | 8,60 | 15,70 | ± | 9,50 | 8,53 | ± | 1,28 |
| Kooy (2009) (82) | Biguanide | Placebo | 3,40 | 390 | 1326 |  | 58,6 | 64,00 |  | 10,00 | 7,00 | ± | 8,00 | 7,90 | ± | 1,20 |
| Kothny (2012) (83) | DPP4i | Placebo | 0,46 | 449 | 207 | 53,5 | 48,0 | 59,10 |  | 10,10 | 13,20 |  | 7,90 | 8,80 |  | 1,00 |
| Kovacs (2015) (84) | SGLT2i | Placebo | 1,40 | 498 | 697 | 36,4 | 55,8 | 54,60 | ± | 10,50 |  |  |  | 8,16 | ± | 0,92 |
| LEAD-3 Mono (85) | GLP1A | Sulfonylurea | 0,96 | 746 | 716 | 77,0 | 54,0 | 53,40 |  | 10,90 | 5,60 |  | 5,10 | 8,40 |  | 1,20 |
| LEADER (86) | GLP1A | Placebo | 3,80 | 9340 | 35492 | 77,5 | 36,0 | 64,40 | ± | 7,20 | 12,90 | ± | 8,10 | 8,70 | ± | 1,50 |
| LIRA-RENAL (87) | GLP1A | Placebo | 0,50 | 277 | 139 | 94,2 | 52,6 | 66,30 | ± | 8,00 | 14,20 | ± | 7,50 | 8,00 | ± | 0,85 |
| LIRA-SWITCH (88) | GLP1A | DPP4i | 0,50 | 406 | 203 |  | 39,0 | 56,50 | ± | 9,70 | 7,60 | ± | 6,20 | 8,20 | ± | 0,89 |
| Laakso (2015) (89) | DPP4i | Sulfonylurea | 1,00 | 241 | 241 |  | 35,2 | 65,90 | ± | 9,40 |  |  |  | 8,03 | ± | 0,94 |
| Lavalle-Gonzalez (2013) (90) | SGLT2i | Placebo | 0,50 | 918 | 459 | 70,5 | 53,0 | 55,30 |  | 9,80 | 6,80 |  | 5,30 | 8,00 |  | 0,90 |
| Ledesma (2019) (91) | DPP4i | Placebo | 0,44 | 302 | 133 | 55,6 | 39,1 | 72,30 |  | 5,10 |  |  |  | 8,20 |  | 0,80 |
| Leiter (2014) (92) | SGLT2i | Placebo | 0,96 | 962 | 924 | 84,0 | 33,0 | 63,60 | ± | 7,00 | 13,00 | ± | 8,40 | 8,10 | ± | 0,80 |
| Leiter (2015) (93) | SGLT2i | Sulfonylurea | 2,00 | 1450 | 2900 | 66,8 | 45,4 | 56,30 | ± | 9,00 | 6,60 | ± | 5,00 | 7,80 | ± | 0,80 |
| Lewin (2015)-1 (94) | SGLT2i+DPP4i | DPP4i | 1,00 | 402 | 402 | 77,4 | 43,6 | 53,80 | ± | 11,50 |  |  |  | 8,05 | ± | 0,89 |
| Lewin (2015)-2 (94) | SGLT2i | DPP4i | 1,00 | 398 | 398 | 77,4 | 43,6 | 53,80 | ± | 11,50 |  |  |  | 8,05 | ± | 0,89 |
| Marbury (1999) (95) | Glinide | Sulfonylurea | 0,96 | 544 | 522 | 79,0 | 34,0 | 58,70 |  | 9,00 | 8,30 |  | 6,80 | 8,90 |  | 1,60 |
| Marre (2002) (96) | Glinide+Biguanide | Placebo | 0,44 | 467 | 205 | 90,8 | 38,7 | 57,60 | ± | 9,19 | 7,00 | ± | 6,00 | 8,17 | ± | 0,98 |
| Mathieu (2016) (97) | SGLT2i+DPP4i | Placebo | 1,00 | 320 | 320 | 91,9 | 52,5 | 55,00 | ± | 9,60 | 8,00 | ± | 6,60 | 8,17 | ± | 0,98 |
| Matthaei (2016) (98) | DPP4i | Placebo | 1,00 | 315 | 315 | 87,0 | 53,1 | 54,50 |  | 9,30 | 7,40 |  | 5,80 | 7,86 |  | 0,93 |
| Matthews (2010) (99) | DPP4i | Sulfonylurea | 0,96 | 3118 | 2993 | 86,3 | 46,1 | 57,50 | ± | 9,19 | 5,70 | ± | 5,00 | 7,30 | ± | 0,70 |
| Matthews (2019) (100) | DPP4i | Placebo | 5,00 | 2001 | 10005 | 61,0 | 51,3 | 54,60 | ± | 9,20 | 0,28 | ± | 0,49 | 6,70 | ± | 0,50 |
| Mazzone (2006) (101) | TZD | Sulfonylurea | 1,50 | 458 | 687 | 65,4 | 36,9 | 59,60 | ± | 8,20 | 7,75 | ± | 6,80 | 7,41 | ± | 0,97 |
| Merker (2015) (102) | SGLT2i | Placebo | 1,40 | 637 | 892 | 54,6 | 44,0 | 56,00 | ± | 9,70 |  |  |  | 7,90 | ± | 0,70 |
| Moses (2015) (103) | DPP4i | SGLT2i | 1,00 | 422 | 422 | 41,5 | 53,8 | 55,40 | ± | 10,20 | 8,00 | ± | 5,35 | 8,40 | ± | 0,90 |
| Muller-Wieland (2018)-1 (104) | SGLT2i | Sulfonylurea | 0,96 | 627 | 602 | 99,0 | 33,5 | 58,60 | ± | 8,40 | 6,70 | ± | 5,10 | 8,30 | ± | 0,80 |
| Muller-Wieland (2018)-2 (104) | SGLT2i+DPP4i | Sulfonylurea | 0,96 | 625 | 600 | 99,0 | 33,5 | 58,60 | ± | 8,40 | 6,70 | ± | 5,10 | 8,30 | ± | 0,80 |
| Muller-Wieland (2018)-3 (104) | SGLT2i | Sulfonylurea | 0,96 | 939 | 901 |  | 33,5 | 58,60 | ± | 8,40 | 6,70 | ± | 5,10 | 8,30 | ± | 0,80 |
| Nauck (2016)-1 (105) | DPP4i+TZD | TZD | 0,63 | 801 | 501 | 85,0 | 47,0 | 56,60 | ± | 10,96 |  |  |  | 8,13 | ± | 0,90 |
| Nauck (2016)-2 (105) | TZD | DPP4i | 0,63 | 527 | 329 | 82,0 | 43,0 | 56,00 | ± | 10,40 |  |  |  | 8,00 | ± | 0,90 |
| Nissen (2008) (106) | TZD | Sulfonylurea | 1,50 | 543 | 815 | 80,6 | 34,1 | 59,70 | ± | 9,10 |  |  |  | 7,40 | ± | 1,00 |
| Olansky (2011) (107) | DPP4i | Biguanide | 0,81 | 1246 | 1009 | 79,0 | 43,0 | 50,00 | ± | 10,50 | 3,20 | ± | 4,30 | 9,80 | ± | 1,80 |
| PIONEER-3 (108) | GLP1A | DPP4i | 1,40 | 1863 | 2608 | 71,3 | 49,0 | 58,00 | ± | 10,00 | 8,80 | ± | 6,00 | 8,30 | ± | 0,90 |
| PIONEER-5 (109) | GLP1A | Placebo | 0,50 | 324 | 162 | 94,0 | 55,0 | 70,00 |  | 8,00 | 13,90 |  | 7,40 | 7,90 |  | 0,70 |
| PIONEER-6 (110) | GLP1A | Placebo | 1,30 | 3183 | 4138 | 72,4 | 31,4 | 66,00 | ± | 7,00 | 15,10 | ± | 8,50 | 8,20 | ± | 1,60 |
| PIONEER-8 (111) | GLP1A | Placebo | 0,96 | 731 | 702 | 53,3 | 42,9 | 60,00 | ± | 10,00 | 14,80 | ± | 7,90 | 8,20 | ± | 0,70 |
| Perez (2010)-2 (112) | TZD | Biguanide | 0,50 | 399 | 200 |  | 53,0 | 53,70 | ± | 12,00 |  |  |  | 8,65 | ± | 0,07 |
| Perez-Monteverde (2011) (113) | DPP4i | TZD | 0,77 | 492 | 379 | 55,2 | 41,3 | 51,70 |  | 10,10 | 3,50 |  | 3,70 | 9,10 |  | 1,40 |
| Perkovic (2019) (114) | SGLT2i | Placebo | 2,62 | 4401 | 11531 | 65,7 | 33,3 | 63,20 | ± | 9,20 | 16,00 | ± | 8,60 | 8,30 | ± | 1,30 |
| Pfutzner (2011-A) (115) | DPP4i | Placebo | 1,40 | 1304 | 1826 | 76,5 | 50,3 | 51,80 | ± | 10,70 | 1,70 | ± | 3,10 | 9,40 | ± | 1,30 |
| Pinget (2013) (116) | GLP1A | Placebo | 1,40 | 484 | 678 | 82,0 | 49,0 | 55,30 | ± | 9,50 | 8,10 | ± | 5,60 | 8,10 | ± | 0,80 |
| Pollock (2019) (117) | SGLT2i+DPP4 | SGLT2i | 0,46 | 300 | 138 | 37,9 | 29,7 | 64,70 |  | 8,60 | 17,55 |  | 7,70 | 8,44 |  | 1,00 |
| Pratley (2012) (118) | GLP1 | TZD | 0,44 | 740 | 326 | 85,0 | 52,5 | 56,40 | ± | 9,80 | 8,90 | ± | 6,30 | 8,30 | ± | 0,90 |
| Pratley (2018) (119) | SGLT2i | DPP4i | 1,00 | 745 | 745 | 78,1 | 38,7 | 54,80 | ± | 10,70 | 6,20 | ± | 5,20 | 8,50 | ± | 1,00 |
| REWIND (120) | GLP1A | Placebo | 5,40 | 9901 | 53465 | 75,6 | 46,1 | 66,20 | ± | 6,50 | 10,60 | ± | 7,20 | 7,40 | ± | 1,10 |
| Ridderstrale (2018) (121) | SGLT2i | Sulfonylurea | 4,00 | 1545 | 6180 | 67,0 | 46,0 | 55,70 | ± | 10,40 |  |  |  | 7,92 | ± | 0,86 |
| Roden (2015)-1 (122) | SGLT2i | DPP4i | 1,50 | 671 | 1007 | 34,1 | 36,8 | 55,10 | ± | 9,90 |  |  |  | 7,85 | ± | 0,79 |
| Roden (2015)-2 (122) | SGLT2i | Placebo | 1,50 | 676 | 1014 | 33,0 | 46,1 | 54,90 | ± | 10,90 |  |  |  | 7,91 | ± | 0,78 |
| Rosenstock (2012) (123) | SGLT2i | Placebo | 0,92 | 420 | 386 | 74,3 | 48,9 | 53,50 |  | 11,40 | 5,07 |  | 5,05 | 8,34 |  | 1,00 |
| Rosenstock (2013) (124) | DPP4i | Sulfonylurea | 1,00 | 441 | 441 | 70,3 | 56,2 | 69,80 | ± | 4,07 | 5,94 | ± | 6,28 | 7,45 | ± | 0,63 |
| Rosenstock (2014) (125) | SGLT2i | Placebo | 1,00 | 563 | 563 | 93,0 | 60,0 | 55,30 | ± | 10,10 |  |  |  | 8,33 | ± | 7,90 |
| Rosenstock (2015)-1 (126) | SGLT2i | Placebo | 1,50 | 494 | 741 | 44,0 | 47,0 | 58,10 | ± | 9,40 |  |  |  | 8,20 | ± | 0,80 |
| Rosenstock (2019)-1 (127) | SGLT2i+DPP4i | SGLT2i | 0,46 | 579 | 266 | 88,9 | 47,4 | 55,90 | ± | 10,90 | 7,60 | ± | 6,30 | 8,20 | ± | 0,90 |
| Rosenstock (2019)-2 (127) | SGLT2i+DPP4i | DPP4i | 0,46 | 581 | 267 | 88,7 | 46,7 | 57,00 |  | 9,90 | 7,50 |  | 5,80 | 8,30 |  | 1,00 |
| SAVOR-TIMI 53 (128) | DPP4i | Placebo | 2,10 | 16492 | 34633 | 75,1 | 32,7 | 65,00 | ± | 8,60 | 10,30 | ± | 6,80 | 8,00 | ± | 1,40 |
| SCALE (129) | GLP1A | Placebo | 1,03 | 846 | 871 | 82,5 | 54,2 | 54,70 | ± | 9,80 | 6,70 | ± | 5,07 | 7,90 | ± | 0,80 |
| SUSTAIN-1 (130) | GLP1A | Placebo | 0,57 | 387 | 221 | 60,0 | 46,0 | 53,90 | ± | 11,00 | 4,06 | ± | 5,48 | 7,95 | ± | 0,85 |
| SUSTAIN-2 (131) | GLP1A | DPP4i | 1,04 | 1225 | 1274 | 69,0 | 49,0 | 54,60 |  | 10,40 | 6,60 |  | 5,10 | 8,20 |  | 0,90 |
| SUSTAIN-6 (132) | GLP1A | Placebo | 1,90 | 3297 | 6264 | 82,0 | 41,5 | 64,80 | ± | 7,60 | 14,00 | ± | 8,54 | 8,70 | ± | 1,49 |
| SUSTAIN-8 (133) | GLP1A | SGLT2i | 0,96 | 788 | 756 | 74,0 | 49,0 | 57,50 | ± | 10,70 | 7,20 | ± | 5,40 | 8,20 | ± | 1,00 |
| Scherbaum (2008) (134) | DPP4i | Placebo | 0,96 | 306 | 294 | 99,3 | 40,7 | 62,80 |  | 11,00 | 2,70 |  | 3,20 | 6,80 |  | 0,40 |
| Schernthaner (2004) (135) | TZD | Biguanide | 0,96 | 1194 | 1146 |  | 42,0 | 56,00 |  | 9,30 | 3,10 |  | 3,80 | 8,70 |  | 1,00 |
| Schernthaner (2013) (136) | SGLT2i | DPP4i | 0,96 | 755 | 725 | 63,5 | 43,1 | 56,70 | ± | 9,30 | 9,70 | ± | 6,30 | 8,10 | ± | 0,90 |
| Schernthaner (2015) (137) | DPP4i | Sulfonylurea | 0,96 | 720 | 691 | 98,6 | 36,7 | 72,70 | ± | 5,40 | 7,60 | ± | 6,00 | 7,62 | ± | 0,65 |
| Schweizer (2007) (138) | DPP4i | Biguanide | 1,00 | 780 | 780 | 69,7 | 42,5 | 53,60 |  | 10,20 | 1,03 |  |  | 8,70 |  | 1,10 |
| Schweizer (2009) (139) | DPP4i | Biguanide | 0,46 | 335 | 154 | 70,5 | 47,0 | 70,20 | ± | 5,10 | 3,00 | ± | 4,70 | 7,70 | ± | 0,60 |
| Scott (140) | DPP4i | SGLT2i | 0,46 | 613 | 282 | 78,2 | 45,0 | 67,70 |  | 8,50 | 10,50 |  | 7,00 | 7,70 |  | 0,70 |
| Seck (2010) (141) | DPP4i | Sulfonylurea | 2,00 | 1172 | 2344 | 74,3 | 38,7 | 56,60 | ± | 9,80 | 6,20 | ± | 5,40 | 7,60 | ± | 0,90 |
| Seino (2010) (142) | GLP1A | Sulfonylurea | 0,46 | 400 | 184 |  | 35,0 | 58,50 | ± | 10,40 | 8,50 | ± | 6,80 | 8,78 | ± | 0,97 |
| Seino (2016) (143) | GLP1A+Insulin | Insulin+Placebo | 0,69 | 257 | 177 |  | 42,3 | 59,80 | ± | 11,30 | 14,69 | ± | 8,60 | 8,80 | ± | 0,90 |
| Stenlof (2013) (144) | SGLT2i | Placebo | 0,50 | 584 | 292 | 69,8 | 54,2 | 55,70 |  | 10,90 | 4,20 |  | 4,10 | 8,00 |  | 1,00 |
| Strain (2013) (145) | DPP4i | Placebo | 0,46 | 278 | 128 | 96,4 | 61,9 | 74,40 |  | 4,00 | 10,60 |  | 6,90 | 7,90 |  | 0,70 |
| Strojek (2014) (146) | SGLT2i | Placebo | 0,90 | 592 | 533 |  | 51,0 | 60,30 | ± | 10,20 | 7,40 | ± | 5,70 | 8,15 | ± | 0,80 |
| TECOS (147) | DPP4i | Placebo | 3,00 | 14671 | 44013 | 68,2 | 29,5 | 65,50 | ± | 8,00 | 11,60 | ± | 8,10 | 7,20 | ± | 0,50 |
| Tolman (2009) (148) | TZD | Sulfonylurea | 3,00 | 2097 | 6291 | 62,1 | 44,5 | 55,00 |  |  | 5,40 |  |  | 9,50 | ± | 2,00 |
| UKPDS 33 – 1 (149) | Sulfonylurea | Placebo | 10,00 | 1515 | 15150 | 83,0 | 38,0 | 54,00 |  | 9,00 | 0,00 | ± | 0,00 | 6,20 |  |  |
| UKPDS 33 – 2 (149) | Sulfonylurea | Placebo | 10,00 | 1511 | 15110 | 83,0 | 38,0 | 54,00 |  | 9,00 | 0,00 | ± | 0,00 | 6,20 |  |  |
| UKPDS 33 – 3 (149) | Insulin | Placebo | 10,00 | 1807 | 18070 | 83,0 | 38,0 | 54,00 |  | 9,00 | 0,00 | ± | 0,00 | 6,20 |  |  |
| Wainstein (2011) (150) | TZD | DPP4i+ Biguanide | 0,61 | 517 | 315 | 64,4 | 45,2 | 52,40 | ± | 10,70 | 3,20 | ± | 4,00 | 9,00 | ± | 1,30 |
| Wang (2017) (151) | DPP4i | Placebo | 0,46 | 380 | 175 | 10,1 | 48,7 | 57,80 |  | 9,50 | 8,20 |  | 5,70 | 8,10 |  | 0,90 |
| Watada (2019) (152) | GLP1 | Placebo | 0,48 | 210 | 101 |  | 33,3 | 56,60 |  | 10,40 | 14,33 |  | 7,79 | 8,61 |  | 0,88 |
| Wilding (2012) (153) | SGLT2i | Placebo | 0,90 | 800 | 720 | 96,4 | 50,8 | 58,80 | ± | 8,60 | 13,50 | ± | 7,30 | 8,47 | ± | 0,77 |
| Wilding (2013) (154) | SGLT2i | Placebo | 1,00 | 469 | 469 | 82,1 | 51,3 | 56,80 | ± | 8,30 | 10,30 | ± | 6,70 | 8,10 | ± | 0,90 |
| Yale (2014) (155) | SGLT2i | Placebo | 1,00 | 269 | 269 | 86,7 | 36,7 | 68,20 | ± | 8,40 | 16,40 | ± | 10,10 | 8,00 | ± | 0,90 |
| Yang (2015) – 1 (156) | DPP4i | Placebo | 0,46 | 279 | 128 |  | 41,90 | 58,70 |  | 9,32 | 6,90 |  | 4,07 | 8,70 |  | 0,98 |
| Yang (2018) (157) | SGLT2i | Placebo | 0,46 | 272 | 125 | 0,0 | 51,9 | 58,60 | ± | 8,90 | 12,20 | ± | 6,70 | 8,58 | ± | 0,81 |

Continuous data are presented as mean ± SD.

RCTs in red the studies with at least 1% of overall weight, which is based on the ratio between each individual study exposure (in patient-years) vs overall exposure.

Most of large RCTs are noted as their acronyms and other studies are annotated as First author (publication year).

The presence of dash followed by a number (e.g. Rosenstock (2015)-1) denotes that one of multiple study arms is included.

This table excluded studies or study arms in which no clinical event occurred in the active or in the control arm.

Patients were randomized to active treatment (DPP4i [46 trials] or DPP4i + TZD [2 trials] or GLP1A [47 trials] or GLP1A + Insulin [6 trials] or Glinide [1 trial] or Glinide + Metformin [2 trials] or Metformin [2 trials] or SGLT2i [39 trials] or SGLT2i + DPP4i [8 trials] or SGLT2i + GLP1A [2 trials] or Sulfonylurea [4 trials] or TZD [13 trials] or TZD + Metformin [1 trial] or TZD + Sulfonylurea [1 trial] or Insulin [1 trial] or Benflurex [1 trial]) vs. control (placebo [85 trials] or DPP4i [18 trials] or DPP4i + Metformin [1 trial] or DPP4i + TZD [1 trial] or Insulin [1 trials] or Metformin [9 trials] or Insulin + Placebo [3 trials] or Sulfonylurea [33 trials] or Biguanide + Sulphonylurea [3 trials] or TZD [9 trials] or GLP1A [1 trial] or SGLT2i [10 trials] or GLP1A + Placebo [1 trial] or Glinide [1 trial]).

**Supplementary Table S3.** Risk of bias analysis

**
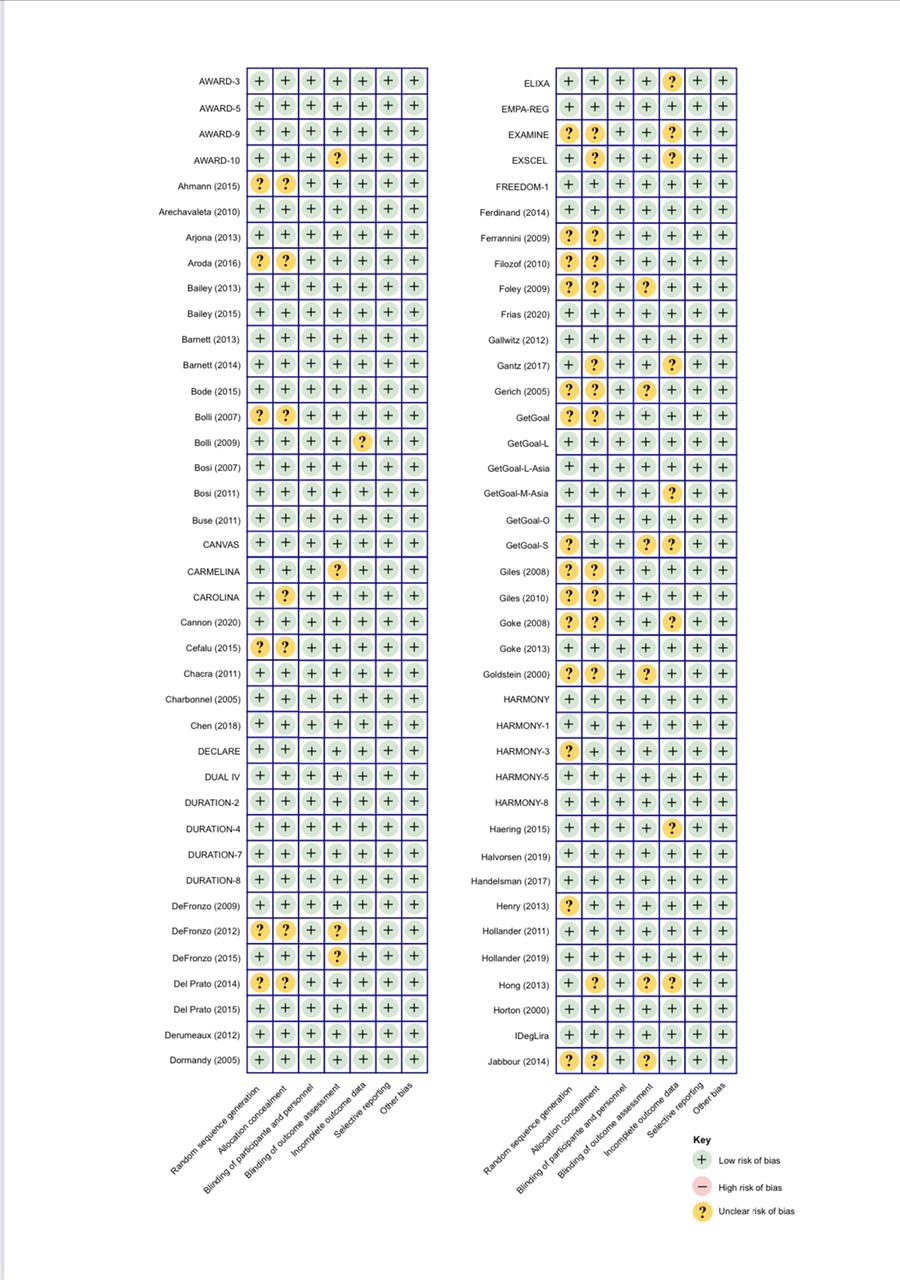
**

**
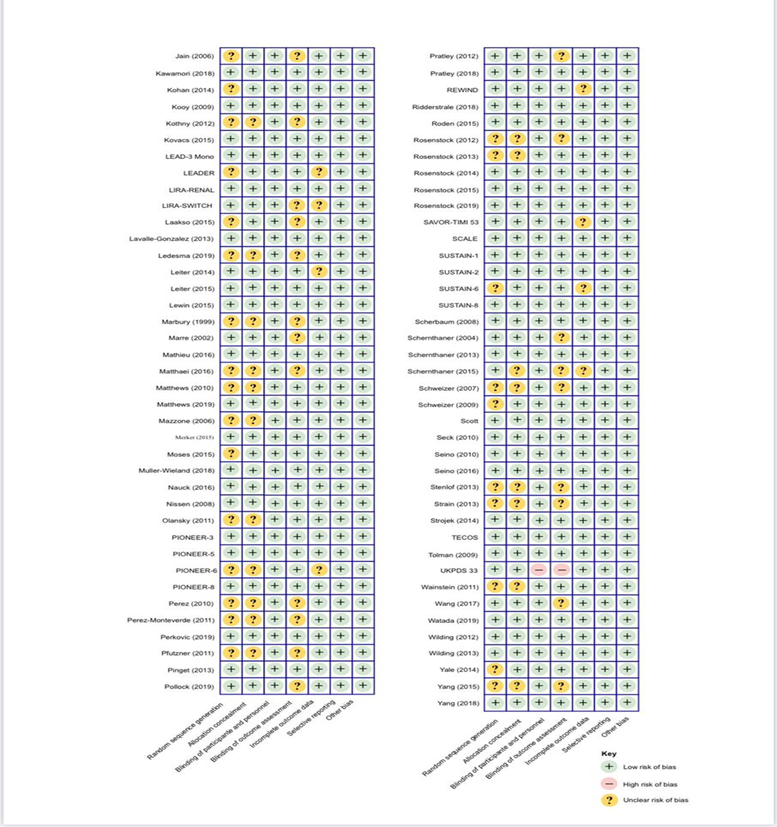
**

**Supplementary Table S4.** Egger's Regression Tests for Funnel Plot Asymmetry

|  | t | z | p-value |
| --- | --- | --- | --- |
| Non-fatal MACE |  |  |  |
| weighted regression with multiplicative dispersion | -1.3890 |  | 0.1668 |
| mixed-effects meta-regression model |  | -1.1414 | 0.2537 |
| All-cause death |  |  |  |
| weighted regression with multiplicative dispersion | -0.7712 |  | 0.4414 |
| mixed-effects meta-regression model |  | 0.4914 | 0.6231 |
| Hospitalization due to heart failure |  |  |  |
| weighted regression with multiplicative dispersion | 0.1854 |  | 0.8532 |
| mixed-effects meta-regression model |  | 0.2333 | 0.8155 |

**Supplementary Table S5:** Baseline data of participants in Brazilian Diabetes Study cohort (outpatient diabetic individuals)

|  | **Overall** | **CVD** | **No CVD** |
| --- | --- | --- | --- |
| n | 1030 | 179 | 851 |
| **Demographics** |  |  |  |
| Age, years | 57±8.1 | 60.2±7.6 | 56.7±8.2 |
| Male | 611 (59,3) | 113 (63,1) | 489 (58,5) |
| Ethnicity (self-declared) |  |  |  |
| White | 708 (67) | 128 (71,5) | 580 (68,2) |
| Pardo | 176 (17,8) | 28 (15,6) | 148 (17,4) |
| Black | 115 (11) | 18 (10,1) | 97 (11,4) |
| Asian | 19 (1,8) | 4 (2,2) | 15 (1,8) |
| Labor status |  |  |  |
| Working | 439 (41,9) | 59 (33) | 380 (44,7) |
| Retired | 163 (15,6) | 38 (21,2) | 125 (14,7) |
| Unemployed | 48 (4,6) | 6 (3,4) | 42 (4,9) |
| **Medical history** |  |  |  |
| Time with diabetes, years | 9,7±7,4 | 12,2±9,5 | 9,2±6,8 |
| Hypertension, % | 838 (81,4) | 175 (97,8) | 663 (77,9) |
| Dyslipidemia, % | 764 (74,2) | 153 (85,5) | 611 (71,8) |
| CVD, % | 179 (17,4) |  | na |
| CHD, % | 155 (15) | 155 (86,6) |  |
| *Stable CAD* | 44 (4,3) | 44 (24,6) |  |
| *PCI or CAGB* | 76 (7,4) | 76 (42,5) |  |
| Stroke, % | 21 (2) | 21 (11,7) |  |
| PAD, % | 11 (1,1) | 11 (6,1) |  |
| Smoker, % | 69 (6,7) | 15 (8,4) | 292 (34,3) |
| **Follow-up** |  |  |  |
| Follow-up time, years | 2.40±0.94 | 2.48±1.15 | 2.38±0.89 |
| MACE, % | 55/1027 (5,4) | 21/176 (11,9) | 34/851 (4) |
| Death, % | 23/1027 (2,2) | 9/ 176 (5,1) | 14/851 (1,6) |
| ACS, % | 14/1027 (1,4) | 9/ 176 (5,1) | 5/ 851 (0,6) |
| PCI_or_CAGB, % | 10/ 1027 (1) | 2/176 (1,1) | 8/851 (0,9) |
| Stroke, % | 8/ 1027 (0,8) | 1/176 (0,6) | 7/851 (0,8) |
| **Antidiabetic therapies, %** |  |  |  |
| Metformin, % | 1015 (98,5) | 176/179 (98,3) | 839/851 (98,6) |
| Sulphonylurea, % | 420 (41,7) | 64/179 (35,8) | 356/851 (41,8) |
| GLP1a, % | 10 (2,6) | 1/179 (0.6) | 9/851 (1,1) |
| Pioglitazone, % | 30 (2,9) | 3/179 (1,7) | 27/851 (3,2) |
| DPP4i, % | 193 (18,7) | 19/179 (10,6) | 174/851 (20,4) |
| SGLT2i, % | 157 (16,6) | 26/179 (14,5) | 131/851 (15,4) |
| Insulin, % | 201 (19,5) | 46/179 (25,7) | 155/851 (18,2) |

Values are presented as mean ± SD or n (%). Hypertension, defined as previous diagnosis or use of any antihypertensive medication; Dyslipidemia, defined by LDL-C>160mg/dL, TG>150mg/dL, HDL<40 (female) or <50mg/dL (male), or use of any lipid lowering drug (statins, fibrates, ezetimibe); CVD, cardiovascular disease (coronary heart disease, cerebrovascular disease or peripheral artery disease); CHD, coronary heart disease (previous ACS, revascularization or diagnosed coronary artery disease); ACS, acute coronary syndrome (unstable angina, myocardial infarction); CAD, coronary artery disease; PCI, percutaneous coronary intervention; CAGB, coronary artery bypass graft; PAD, peripheral artery disease (defined by previous limb amputation, revascularization or diagnosed peripheral artery stenosis); ARB, angiotensin II receptor blocker; CBB, calcium channel blocker; ACEi, angiotensin converter enzyme inhibitor; Sglt2i, sodium-glucose cotransporter type 2 inhibitor; GLP1, glucagon-like peptide 1.

**Supplementary Table S6:** Baseline data of diabetic participants in B-CaRe:QCOR cohort (acute coronary syndromes registry)

|  | **Overall** |
| --- | --- |
| n | 1174 |
| **Demographics** |  |
| Age, years | 63.2±11.5 |
| Male | 632 (54.1) |
|  |  |
| **Medical history** |  |
| Time with diabetes, years | 10,3±7.8 |
| Hypertension, % | 1030 (87.7) |
| Dyslipidemia, % | 876 (74.6) |
| Prior CVD, % | 248 (21.1) |
| CHD, % | 197 (16.8) |
| *Stable CAD* | 57 (4.9) |
| *PCI or CAGB* | 173 (14.7) |
| Stroke, % | 51 (4.3) |
| PAD, % | 24 (2.0) |
| Smoker, % | 336 (28.6) |
|  |  |
| **Antidiabetic therapies prior to ACS, %** |  |
| Metformin, % | 1009 (85.9) |
| Sulphonylurea, % | 505 (43.0) |
| GLP1a, % | 0 |
| Pioglitazone, % | 13 (1.1) |
| DPP4i, % | 28 (2.4) |
| SGLT2i, % | 0 |
| Insulin, % | 338 (28.8) |
|  |  |
| **Follow-up** |  |
| Follow-up time, years | 5.94±1.9 |
| Non-fatal MACE, % | 184 (15.7) |
| Death, % | 174 (14.8) |
| ACS, % | 129 (11.0) |
| PCI_or_CAGB, % | 239 (20.4) |
| Stroke, % | 55 (4.7) |

Values are presented as mean ± SD or n (%). Hypertension, defined as previous diagnosis or use of any antihypertensive medication; Dyslipidemia, defined by LDL-C>160mg/dL, TG>150mg/dL, HDL<40 (female) or <50mg/dL (male), or use of any lipid lowering drug (statins, fibrates, ezetimibe); CVD, cardiovascular disease (coronary heart disease, cerebrovascular disease or peripheral artery disease); ACS, acute coronary syndrome (unstable angina, myocardial infarction); CAD, coronary artery disease; PCI, percutaneous coronary intervention; CAGB, coronary artery bypass graft; PAD, peripheral artery disease (defined by previous limb amputation, revascularization or diagnosed peripheral artery stenosis).

**Supplementary Figure S1**. Study network

(a) Non-fatal MACE

(b) All-cause death

(c) Hospitalizations due to heart failure

The study network presents sharp connections between placebo vs GLP1A, placebo vs SGLT2i, placebo vs DPP4i and placebo vs TZD (pioglitazone). The nodes for Sulfonylurea, TZD, DPP4i, GLP1A, SGLT2i are also tightly connected with most treatment arms, however, it is possible to observe a poor connection between drug combinations (SGLT2i+GLP1A, GLP1A1+Insulin, SGLT2i+DPP4i, GLP1A+TZD, etc) and other treatment arms. By using additive component network meta-analysis, it is possible to account for both single treatments and combinations, but the combinations may be more prone to depend on the effect of indirect comparisons if paralleled to single treatment comparisons.

**Supplementary Figure S2.** Evaluation of publication bias in funnel plots for (a) Non-fatal MACE; (b) All-cause deaths; (c) Hospitalization due to heart failure

(a)

(b)

(c)

**Supplementary Figure S3.** Forest plot comparing antidiabetic therapies for the occurrence of non-fatal major cardiovascular adverse events (MACE) in a (a) non-additive and (b) additive effects network meta-analysis with a random-effects model.

(a)
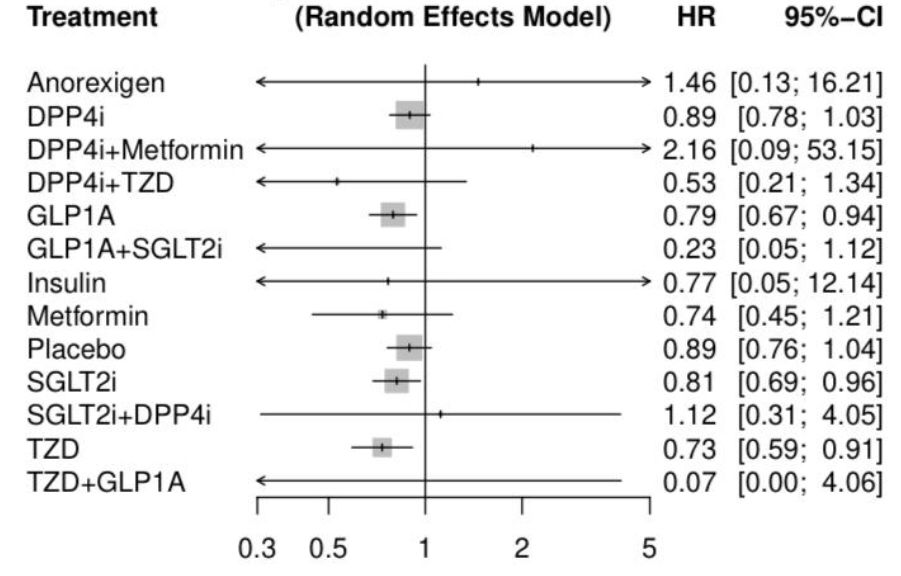


(b)
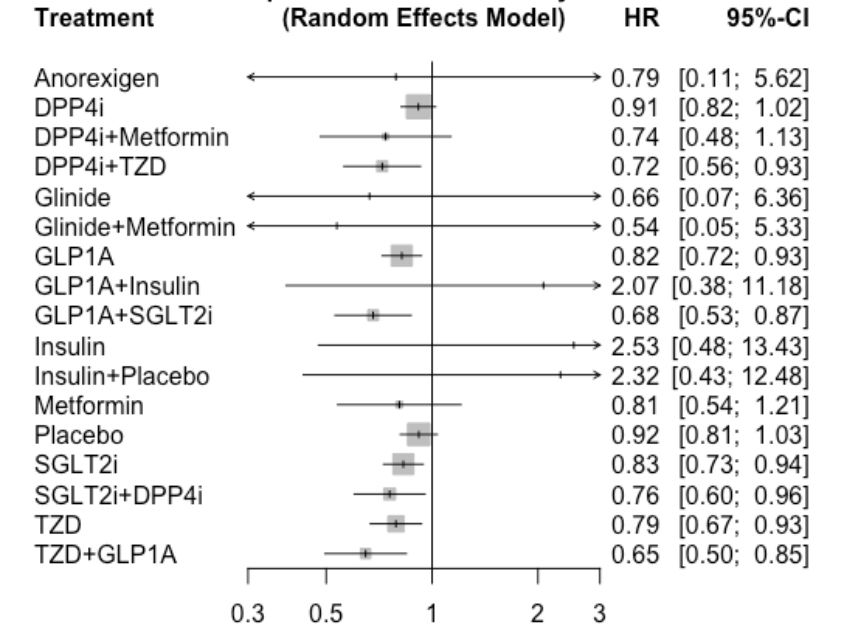


The reference group was considered as sulfonylurea. Number of pairwise comparisons was 118 (105 studies with >100 individuals per arm), number of treatments (n = 14), number of designs (n=37) with 8 active components. Heterogeneity / inconsistency analysis showed tau^2^ = 0; tau = 0; I^2^ = 0% [0.0%; 0.0%]; Q = 96.49 (p=0.9927). Among TZDs (thiazolidinediones) only pioglitazone was included.

**Supplementary Figure S4.** Forest plot comparing antidiabetic therapies for the occurrence of all-cause death in a (a) non-additive and (b) additive effects network meta-analysis with a random-effects model.

(a)
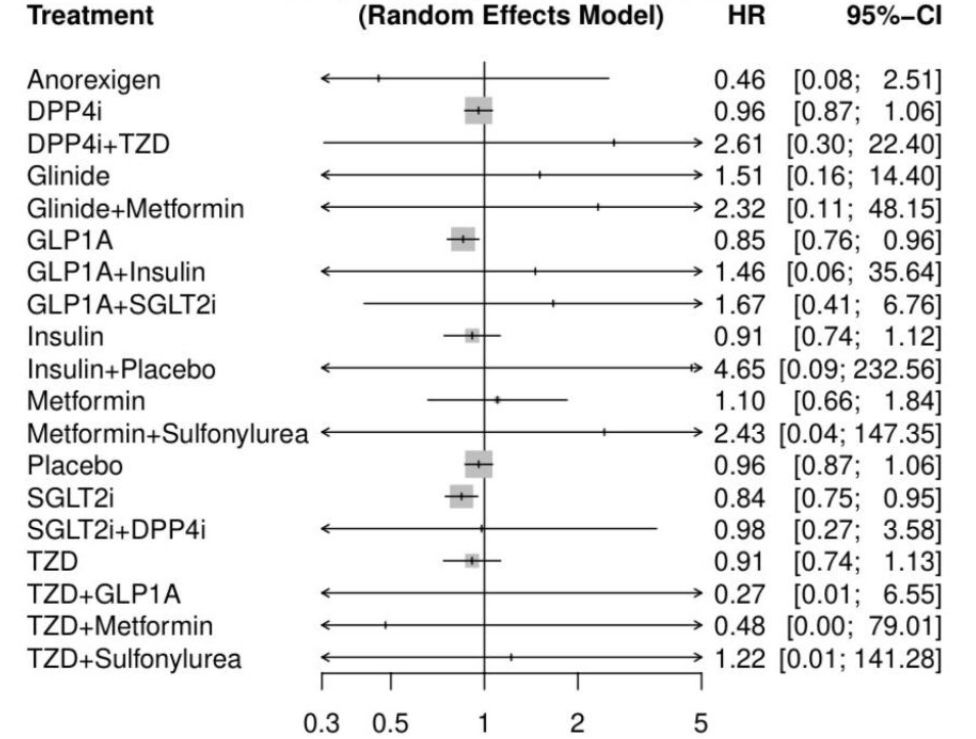


(b)
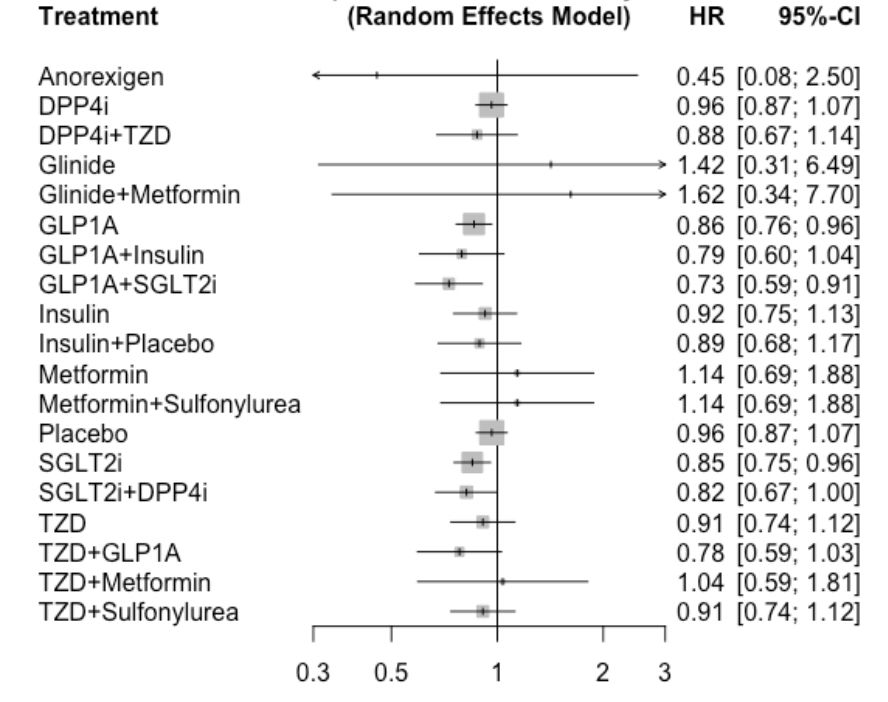


The reference group was considered as sulfonylurea. Number of pairwise comparisons was 140 (127 studies with >100 individuals per arm), number of treatments (n = 20), number of designs (n=35) with 9 active components. Heterogeneity / inconsistency analysis showed tau^2^ = 0; tau = 0; I^2^ = 0% [0.0%; 0.0%]; Q = 82.37 (p=0.9970). Among TZDs (thiazolidinediones) only pioglitazone was included.

**Supplementary Figure S5.** Forest plot comparing antidiabetic therapies for hospitalizations due to heart failure (HHF) in a (a) non-additive and (b) additive effects network meta-analysis with a random-effects model.

(a)
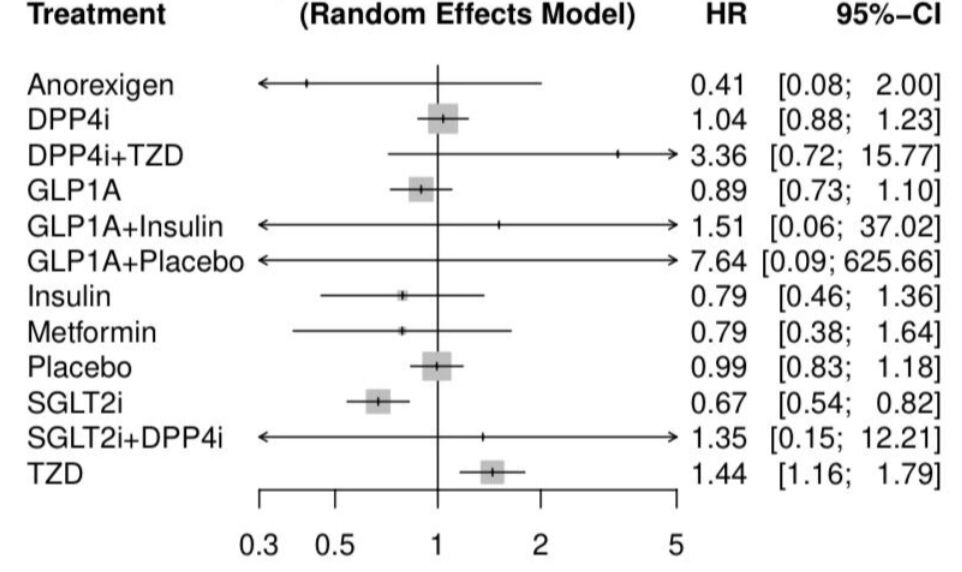


(b)
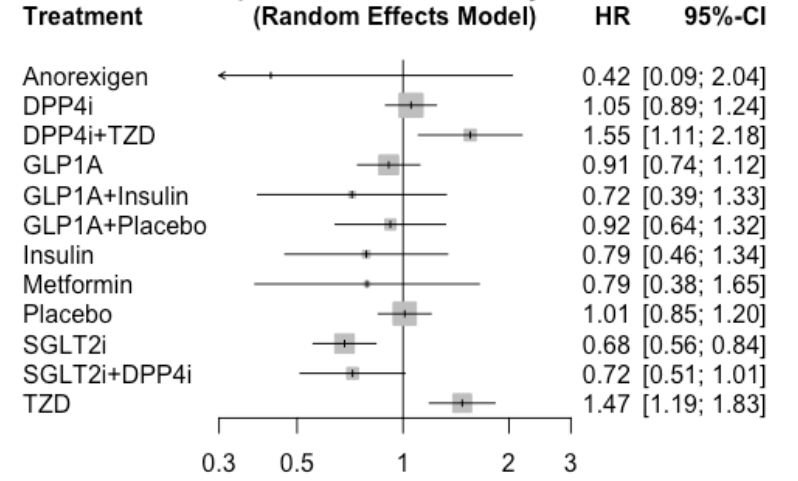


The reference group was considered as sulfonylurea. Number of pairwise comparisons was 65 (55 studies with >100 individuals per arm), number of treatments (n = 13), number of designs (n=21) with 8 active components. Heterogeneity / inconsistency analysis showed tau^2^ = 0; tau = 0; I^2^ = 0% [0.0%; 0.0%]; Q = 43.96 (p=0.8072). Among TZDs (thiazolidinediones) only pioglitazone was included.

**Supplementary Figure S6.** One-way sensitivity analyses for (a to d) the scenario where individual depart from state A (asymptomatic, primary prevention) and for (e to h) the scenario where individual depart from state C (recent acute coronary syndrome or stroke, secondary prevention)

***a and b.*** one-way sensitivity analysis considering 5% discount rate for costs in each add-on therapy in primary prevention


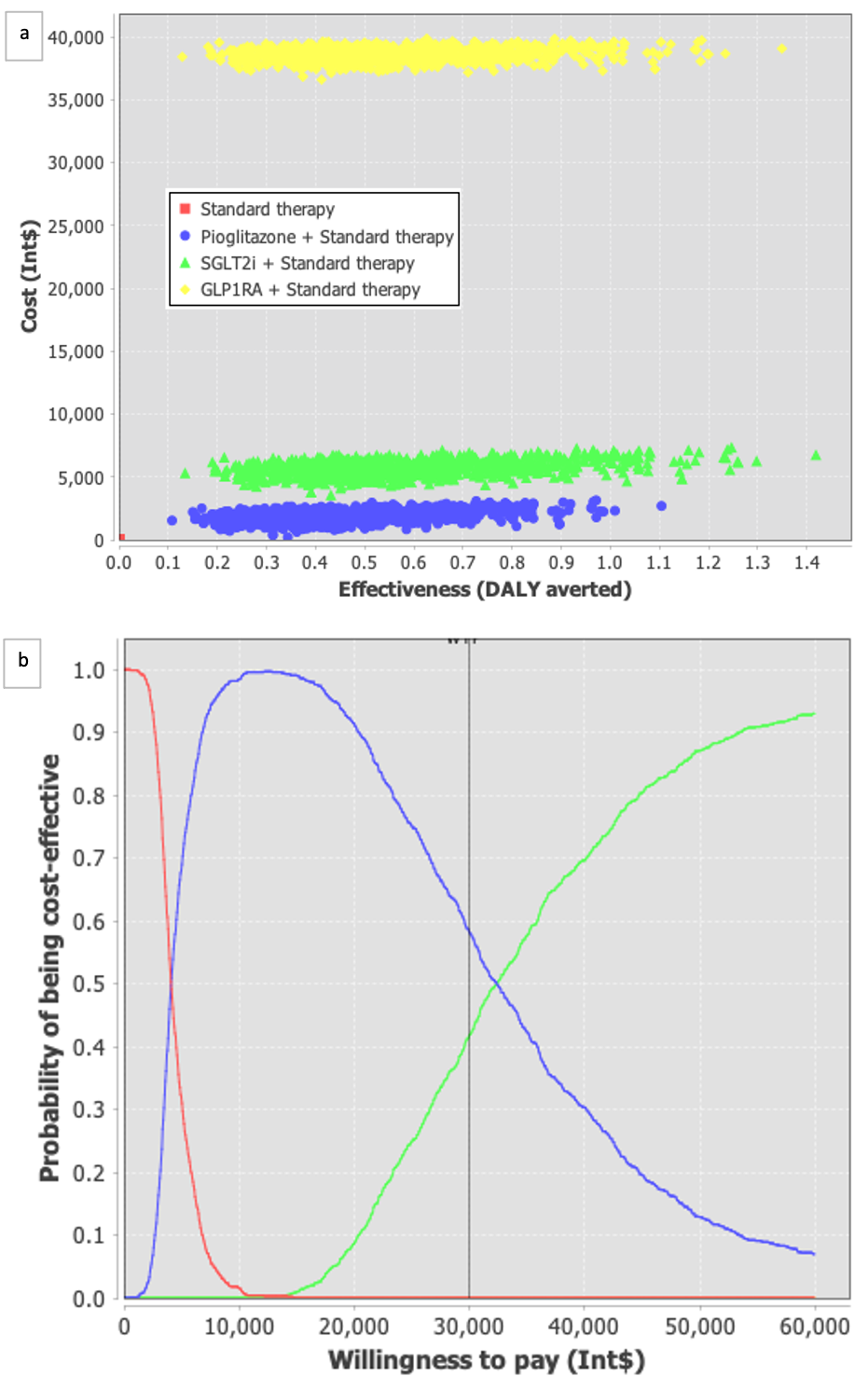


***c and d.*** one-way sensitivity analysis considering 5% discount rate for effectiveness in each add-on therapy in primary prevention


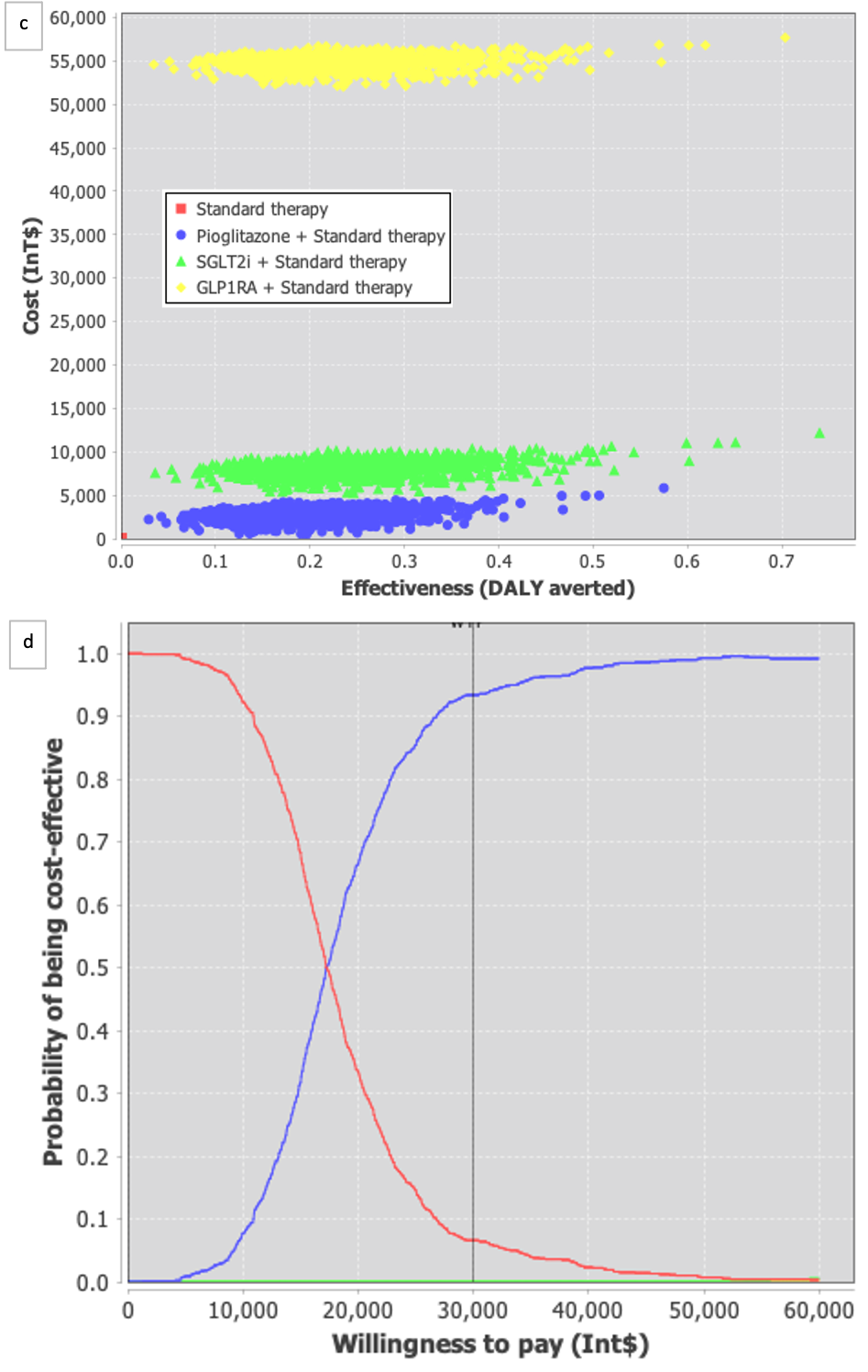


***e and f.*** one-way sensitivity analysis considering 5% discount rate for costs in each add-on therapy in secondary prevention


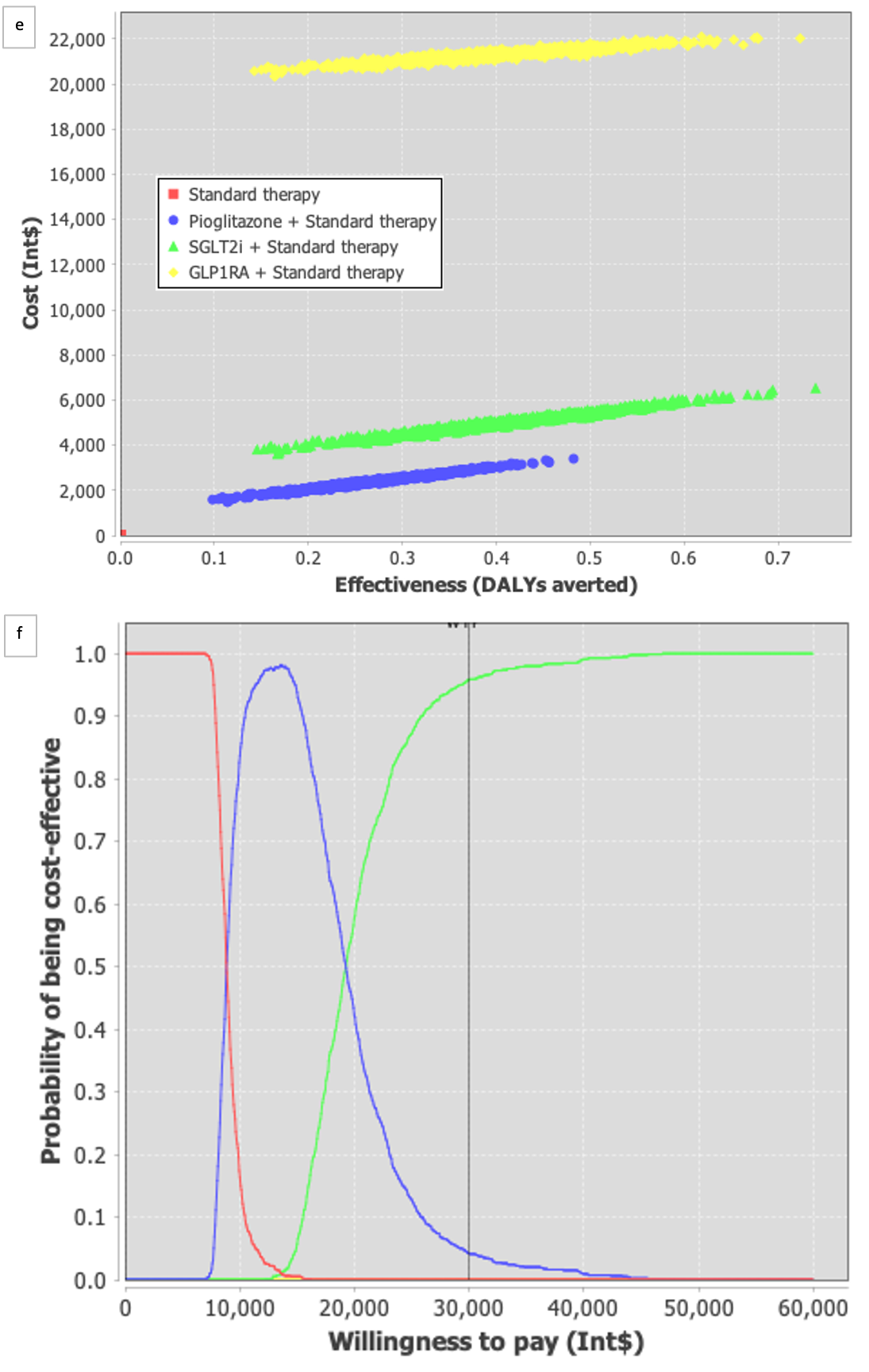


***g and h.*** one-way sensitivity analysis considering 5% discount rate for effectiveness in each add-on therapy in secondary prevention;

***
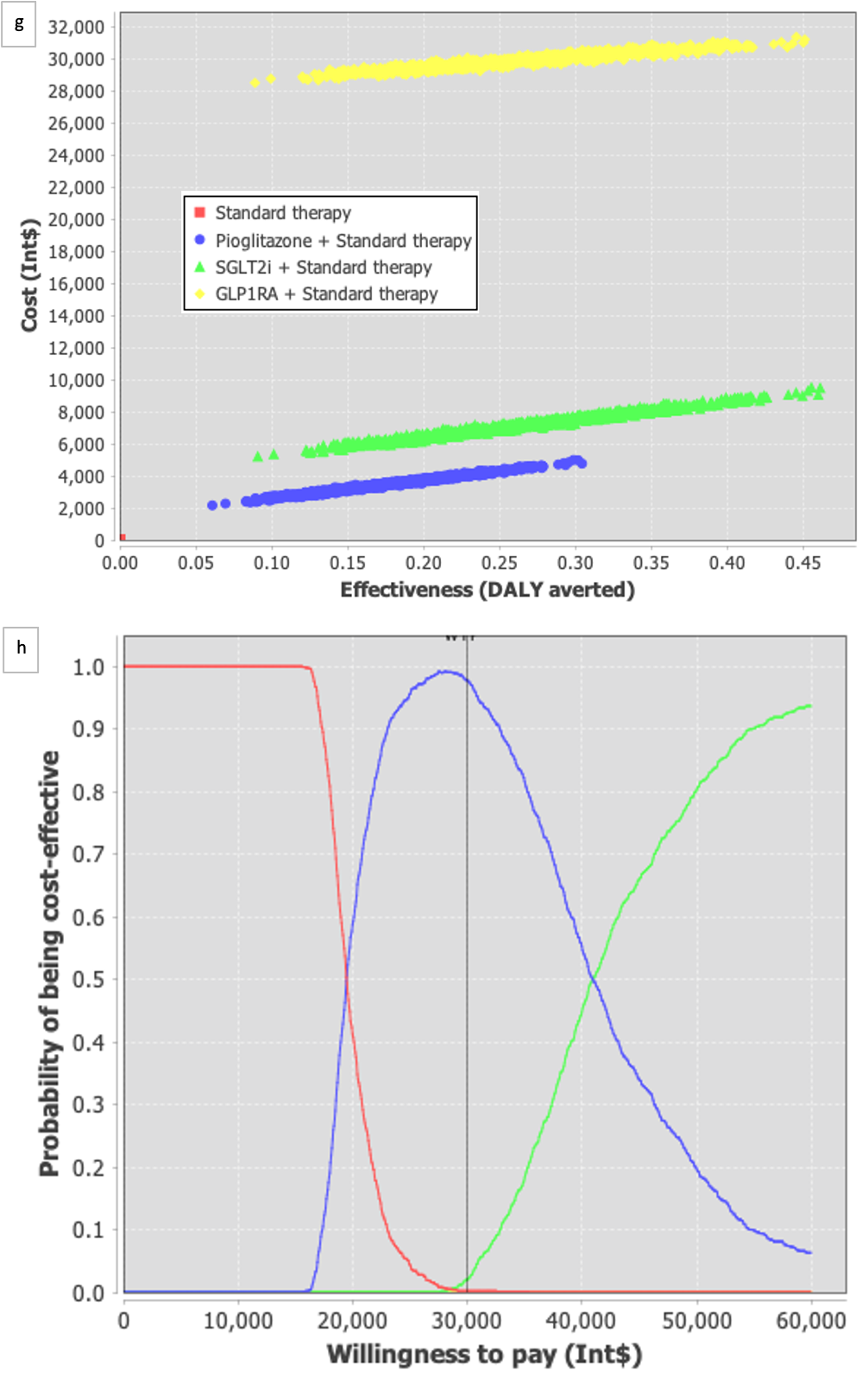
***
